# Contextualizing protein representations using deep learning on protein networks and single-cell data

**DOI:** 10.1101/2023.07.18.549602

**Published:** 2023-07-19

**Authors:** Michelle M. Li, Yepeng Huang, Marissa Sumathipala, Man Qing Liang, Alberto Valdeolivas, Ashwin N. Ananthakrishnan, Katherine Liao, Daniel Marbach, Marinka Zitnik

**Affiliations:** 1 Harvard Medical School, Boston, MA, USA; 2 Roche Pharma Research and Early Development, Pharmaceutical Sciences, Roche Innovation Center Basel, Basel, Switzerland; 3 Division of Gastroenterology, Massachusetts General Hospital, Boston, MA, USA; 4 Division of Rheumatology, Inflammation, and Immunity, Brigham and Women’s Hospital, Boston, MA, USA; 5 Kempner Institute for the Study of Natural and Artificial Intelligence, Harvard University, MA, USA; 6 Broad Institute of MIT and Harvard, Cambridge, MA, USA; 7 Harvard Data Science Initiative, Cambridge, MA, USA

## Abstract

Understanding protein function and discovering molecular therapies require deciphering the cell types in which proteins act as well as the interactions between proteins. However, modeling protein interactions across diverse biological contexts, such as tissues and cell types, remains a significant challenge for existing algorithms. We introduce Pinnacle, a flexible geometric deep learning approach that is trained on contextualized protein interaction networks to generate context-aware protein representations. Leveraging a human multi-organ single-cell transcriptomic atlas, Pinnacle provides 394,760 protein representations split across 156 cell type contexts from 24 tissues and organs. Pinnacle’s contextualized representations of proteins reflect cellular and tissue organization and Pinnacle’s tissue representations enable zero-shot retrieval of the tissue hierarchy. Pretrained Pinnacle protein representations can be adapted for downstream tasks: to enhance 3D structure-based protein representations (PD-1/PD-L1 and B7-1/CTLA-4) at cellular resolution and to study the genomic effects of drugs across cellular contexts. Pinnacle outperforms state-of-the-art, yet context-free, models in nominating therapeutic targets for rheumatoid arthritis and inflammatory bowel diseases, and can pinpoint cell type contexts that are more predictive of therapeutic targets than context-free models (29 out of 156 cell types in rheumatoid arthritis; 13 out of 152 cell types in inflammatory bowel diseases). Pinnacle is a network-based contextual AI model that dynamically adjusts its outputs based on biological contexts in which it operates.

Proteins are the functional units of cells, and their interactions enable different biological functions. The development of high-throughput methods has facilitated the characterization of large maps of protein interactions. Leveraging these protein interaction networks, computational methods [1-6] have been developed to improve the understanding of protein structure [7-13], accurately predict functional annotations [14-19], and inform the design of therapeutic targets [20-23]. Among them, representation learning methods have emerged as a leading strategy to model proteins [24-28]. These approaches can resolve protein interaction networks across tissues [29-33] and cell types by integrating molecular cell atlases [34, 35] and extending our understanding of the relationship between protein and function [36-38]. Protein representation learning methods can predict multicellular functions across human tissues [31], design target-binding proteins [39-42] and novel protein interactions [43], and predict interactions between transcription factors and genes [37, 38].

Proteins can have distinct roles in different biological contexts [44, 45]. While nearly every cell contains the same genome, the expression of genes and the function of proteins encoded by these genes depend on cellular and tissue contexts [29, 46-49]. Gene expression and the function of proteins can also differ significantly between healthy and disease states [47, 50-52]. Therefore, computational methods that incorporate biological contexts can improve the characterization of proteins. However, existing deep learning methods produce protein representations (or embeddings) that are context-free: each protein has only one representation learned from either a single context or an integrated view across many contexts [53-57]. These methods generate one representation for each protein, providing an integrated summary of the protein. While such protein representations can be valuable, they are not tailored to specific biological contexts, such as cell types and disease states. This makes it challenging to use protein representations for predicting molecular phenotypes that vary with cell types as well as for predicting pleiotropy and other protein roles in distinct cell types.

The recent development of sequencing technologies to measure gene expression with single-cell resolution could pave the way toward addressing this challenge. Single-cell transcriptomic atlases [46, 58-62] measure activated genes across a large number of contexts at the resolution of individual cells. Through attention-based deep learning [63-65], which specify models that can pay attention to large inputs and learn the most important elements to focus on in each context, single-cell altases can be leveraged to boost the mapping of gene regulatory networks that drive disease progression and reveal treatment targets [66]. However, incorporating the expression of protein-coding genes into protein interaction networks remains a challenge. Existing algorithms, including protein representation learning, are unable to contextualize protein representations.

We introduce Pinnacle (Protein Network-based Algorithm for Contextual Learning), a self-supervised geometric deep learning model adept at generating protein representations through the analysis of protein interactions within various cellular contexts.^1^ Pinnacle is a contextual AI model for network-based protein representation learning. Leveraging single-cell transcriptomics combined with networks of protein-protein interactions, cell-cell interactions, and a tissue hierarchy, Pinnacle generates high-resolution protein representations tailored to each cell type. In contrast to existing methods that provide a single representation for each protein, Pinnacle generates a distinct representation for each cell type in which a protein-coding gene is activated. With 394,760 contextualized protein representations produced by Pinnacle, where each protein representation is distinctly imbued with cell type specificity, we demonstrate Pinnacle’s capability to integrate protein interactions with the underlying protein-coding gene transcriptomes of 156 cell type contexts, perform transfer learning across cell types, and make predictions for an array of tasks based on the context in which Pinnacle operates. Leveraging Pinnacle, we demonstrate the benefits of contextual artificial intelligence (AI) to infer PD-1/PD-L1 and B7-1/CTLA-4 interactions between cell types and to identify therapeutic targets and nominate cell type contexts for rheumatoid arthritis and inflammatory bowel diseases. Contextual AI models can improve overall performance by dynamically adjusting their outputs based on the context in which they operate. Our results underscore the benefits of contextual AI to understand and interpret biological contexts.

## Results

### Constructing context-aware protein, cell type, and tissue networks.

**Figure 1:**
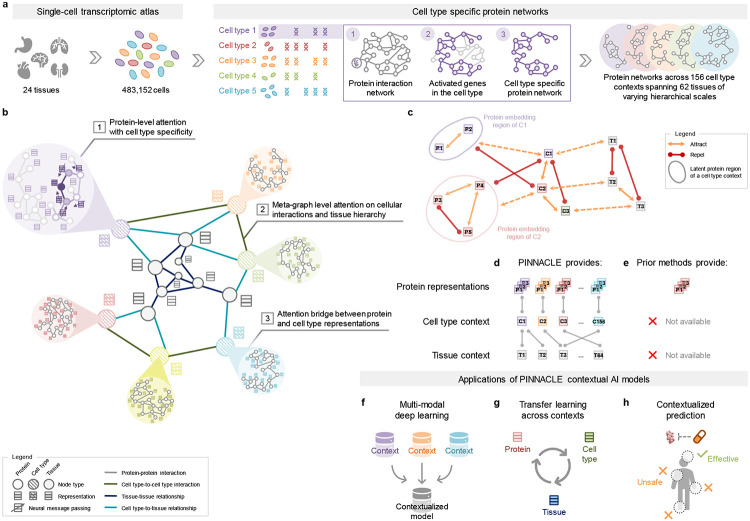
Overview of Pinnacle. (**a**) Cell type-specific protein interaction networks are constructed from a multi-organ single-cell transcriptomic atlas of humans. (**b**) Pinnacle has protein-, cell type-, and tissue-level attention mechanisms that enable the algorithm to generate contextualized representations of proteins, cell types, and tissues in a single unified embedding space. (**c**) Pinnacle is designed such that the nodes (i.e., proteins, cell types, and tissues) that share an edge are embedded closer (attracted) to each other than nodes that do not share an edge (repelled); proteins activated in the same cell type are embedded more closely (attracted) than proteins activated in different cell types (repelled); and cell types are embedded closer to their activated proteins (attracted) than other proteins (repelled). (**d**) As a result, Pinnacle generates protein representations injected with cell type and tissue context; a unique representation is produced for each protein activated in each cell type. Pinnacle simultaneously generates representations for cell types and tissues. (**e**) Existing methods, however, are context-free. They generate a single embedding per protein, representing only one condition or context for each protein, without any notion of cell type or tissue context. (**f-h**) The Pinnacle algorithm and its outputs enable (**f**) multi-modal deep learning (e.g., single-cell transcriptomic data with interactomes), (**g**) transfer learning across contexts (e.g., between proteins, cell types, and tissues), and (**h**) contextualized predictions (e.g., efficacy and safety of therapeutics).

Generating protein representations embedded with cell type context calls for protein interaction networks that consider the same context. We assembled a dataset of context-sensitive protein interactomes, beginning with a multi-organ single-cell transcriptomic atlas [46] that encompasses 24 tissue and organ samples sourced from 15 human donors (Figure 1a). We compile activated genes for every expert-annotated cell type in this dataset by evaluating the average gene expression in cells from that cell type relative to a designated reference set of cells (Figure 1a; Methods 2). Here, ‘activated genes’ are defined as those demonstrating a higher average expression in cells annotated as a particular type than the remaining cells documented in the dataset. Based on these activated gene lists, we extracted the corresponding proteins from the comprehensive reference protein interaction network and retained the largest connected component (Figure 1a). As a result, we have 156 context-aware protein interaction networks, each with 2, 530.5 ± 676.8 proteins, that are maximally similar to the global reference protein interaction network and still highly cell type specific (Supplementary Figure S1). Our context-aware protein interaction networks from 156 cell type contexts span 62 tissues of varying biological scales.

Further, we constructed a network of cell types and tissues (metagraph) to model cellular interactions and the tissue hierarchy (Methods 2). Given the cell type annotations designated by the multi-organ transcriptomic atlas [46], our network consists of 156 cell type nodes. We incorporated edges between pairs of cell types based on the existence of significant ligand-receptor interactions and validated that the proteins correlating to these interactions are enriched in the context-aware protein interaction networks in comparison to a null distribution (Methods 2; Supplementary Figure S2) [67]. Leveraging information on tissues in which the cell types were measured, we began with a set of 24 tissue nodes and established edges between cell type nodes and tissue nodes if the cell type was derived from the corresponding tissue. We then identified all ancestor nodes, including the root, of the 24 tissue nodes within the tissue hierarchy [68] to feature 62 tissue nodes interconnected by parent-child relationships. Our dataset thus comprises 156 context-aware protein interaction networks and a metagraph reflecting cell type and tissue organization.

### Overview of Pinnacle model.

Pinnacle is a self-supervised geometric deep learning model capable of generating protein representations predicated on protein interactions within a spectrum of cell type contexts. Trained on an integrated set of context-aware protein interaction networks, complemented by a network capturing cellular interactions and tissue hierarchy (Figure 1b-c), Pinnacle generates contextualized protein representations that are tailored to cell types in which protein-coding genes are activated (Figure 1d). Unlike context-free models, Pinnacle produces multiple representations for every protein, each contingent on its specific cell type context. Additionally, Pinnacle produces representations of the cell type contexts and representations of the tissue hierarchy (Figure 1d-e). This approach ensures a multifaceted understanding of protein interaction networks, taking into account the myriad of contexts in which proteins function.

Given multi-scale model inputs, Pinnacle learns the topology of proteins, cell types, and tissues by optimizing a unified latent representation space (Figure 1f-g). To infuse the cellular and tissue organization into this embedding space, Pinnacle employs protein-, cell type-, and tissue-level attention along with respective objective functions (Figure 1b-c; Methods 3). Conceptually, pairs of proteins that physically interact (i.e., are connected by edges in input networks) are closely embedded. This ensures that interacting proteins within the same cell type context are situated proximally within the embedding space yet separated from proteins belonging to other cell type contexts. Similarly, proteins should be embedded near their respective cell type contexts while maintaining a substantial distance from unrelated ones (Figure 1c). This approach yields an embedding space that accurately represents the intricacies of relationships between proteins, cell types, and tissues.

Pinnacle disseminates graph neural network messages between proteins, cell types, and tissues using a series of attention mechanisms tailored to each specific node and edge type (Methods 3). The protein-level pretraining tasks consider self-supervised link prediction on protein interactions and cell type masking on protein nodes. These tasks enable Pinnacle to sculpt an embedding space that encapsulates both the topology of the context-aware protein interaction networks and the cell type identity of the proteins. Pinnacle’s cell type- and tissue-specific pretraining tasks rely exclusively on self-supervised link prediction, facilitating the learning of cellular and tissue organization. The topology of cell types and tissues is imparted to the protein representations through an attention bridge mechanism, effectively enforcing tissue and cellular organization onto the protein representations. Pinnacle’s contextualized protein representations capture the structure of context-aware protein interaction networks. The regional arrangement of these contextualized protein representations in the latent space reflects the cellular and tissue organization represented by the metagraph. This leads to a comprehensive and context-specific representation of proteins within a unified cell type and tissue-specific framework.

### Pinnacle’s representations capture cellular and tissue organization.

**Figure 2:**
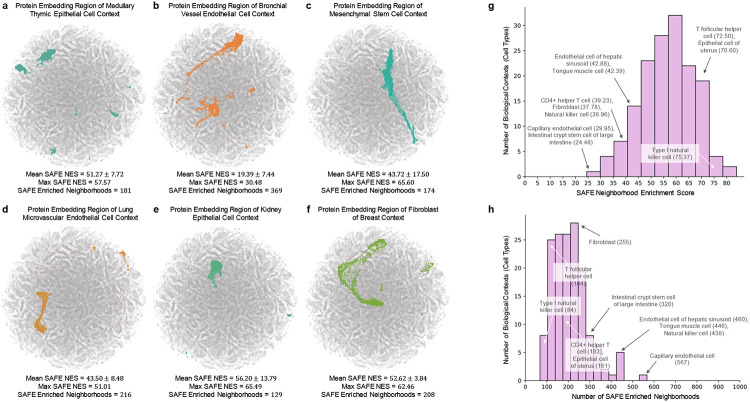
Enrichment of Pinnacle’s protein embedding regions. (**a-f**) Shown are two-dimensional UMAP plots of contextualized protein representations generated by Pinnacle. Each dot is a protein representation, and non-gray colors indicate the cell type context. Gray dots are representations of proteins from other cell types. Each protein embedding region is expected to be enriched neighborhoods that are spatially localized according to cell type context. To quantify this, we compute spatial enrichment of each protein embedding region using SAFE [69], and provide the mean and max neighborhood enrichment scores (NES) and the number of enriched neighborhoods output by the tool (Methods 6 and Supplementary Figure S3). (**g-h**) Distribution of (**g**) the maximum SAFE NES and (**h**) the number of enriched neighborhoods for 156 cell type contexts (each context has *p*-value < 0.05). 10 randomly sampled cell type contexts are annotated, with their maximum SAFE NES or number of enriched neighborhoods in parentheses.

Using context-aware protein interaction networks constructed from a human multi-organ single cell transcriptomic atlas, Pinnacle generates protein representations for each of the 156 cell type contexts spanning 62 tissues of varying hierarchical scales. Each cell type context consists of 2, 530.5 ± 676.8 protein representations (Figure 1a). In total, Pinnacle’s unified multi-scale embedding space comprises 394,760 protein representations, 156 cell type representations, and 62 tissue representations. We show that Pinnacle learns an embedding space of proteins where proteins are localized based on cell type context. We first quantify the spatial enrichment of Pinnacle’s protein embedding regions using a systematic method, SAFE [69] (Methods 6.3). The spatial regions in Pinnacle’s embedding space are significantly enriched for protein representations originating from the same cell type context (Figure 2; Supplementary Figures S3-S4), suggesting that Pinnacle’s contextualized protein representations self-organize meaningfully.

**Figure 3:**
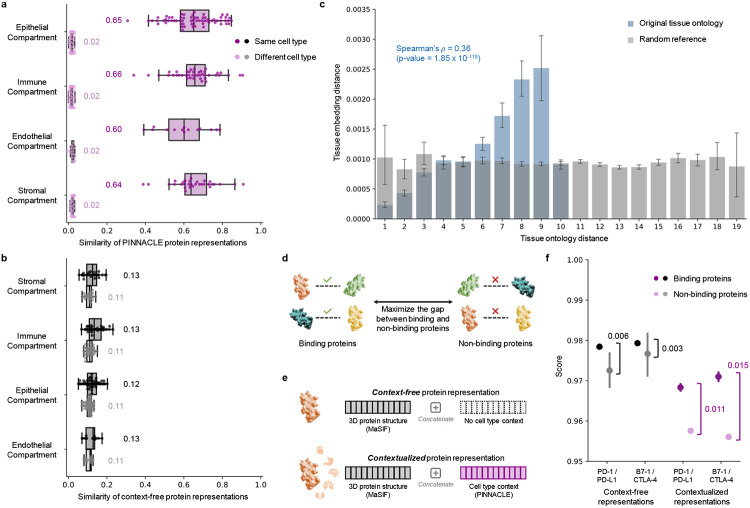
Evaluation of Pinnacle’s contextual representations. (**a-b**) Gap between embedding similarities using (**a**) Pinnacle’s protein representations and (**b**) a non-contextualized model’s protein representations. Similarities are calculated between pairs of proteins in the same cell type (dark shade of color) or different cell types (light shade of color), and stratified by the compartment from which the cell types are derived. Annotations indicate median values. The non-contextualized model is an ablated version of Pinnacle without any notion of tissue or cell type organization (i.e., remove cell type and tissue network and all cell type- and tissue-related components of Pinnacle’s architecture and objective function). (**c**) Embedding distance of Pinnacle’s 62 tissue representations as a function of tissue ontology distance. Gray bars indicate a null distribution (refer to Methods 6 for more details). (**d**) Prediction task in which protein representations are optimized to maximize the gap between binding and non-binding proteins. (**e**) Cell type context (provided by Pinnacle) is injected into context-free structure-based protein representations (provided by MaSIF [10], which learns a protein representation from the protein’s 3D structure) via concatenation to generate contextualized protein representations. Lack of cell type context is defined by an average of Pinnacle’s protein representations. (**f**) Comparison of context-free and contextualized representations in differentiating between binding and non-binding proteins. Scores are computed using cosine similarity. The binding proteins evaluated are PD-1/PD-L1 and B7-1/CTLA-4. Pairwise scores also are calculated for each of these four proteins and proteins that they do not bind with (i.e., RalB, RalBP1, EPO, EPOR, C3, and CFH). The gap between the average scores of binding and non-binding proteins is annotated for context-free and contextualized representations.

Next, we evaluate regions in Pinnacle’s protein latent space to confirm that they are separated according to cell type and tissue contexts. To quantify the quality of Pinnacle’s protein embedding regions, we calculate pairwise similarities of protein representations across cell type contexts. We expect protein representations from the same cell type context to be more similar than those from different cell type contexts. Indeed, the similarities between protein representations from the same cell type are higher than the similarities between protein representations from different cell types (Figure 3a). In contrast, a model without cellular or tissue context fails to capture any differences between protein representations across cell type contexts (Figure 3b). Further, we expect the representations of proteins that act on multiple cell types to be highly dissimilar, reflecting the specialized functions that proteins may have depending on the cell type. We calculate the similarities of protein representations (i.e., cosine similarities of a protein’s representations across cell type contexts) based on the number of cell types that the protein is activated in (Supplementary Figure S5). Proteins’ embedding similarities negatively correlate with the number of cell types in which they act (Spearman’s *ρ* = −0.9798; *p*-value < 0.001). In contrast, protein embedding similarities from a model without cellular or tissue organization have a weaker correlation (Spearman’s *ρ* = −0.6334; *p*-value < 0.001). Such analyses suggest that Pinnacle’s protein embedding regions are organized according to cellular contexts and may capture nuances that are not explicitly defined in the data or model, such as cell type dependent roles of proteins.

In addition to the meaningful separation of Pinnacle’s protein embedding regions, we demonstrate the accuracy of their organization according to the tissue hierarchy. For this evaluation, we directly leverage Pinnacle’s tissue representations to perform zero-shot retrieval of the tissue hierarchy by comparing tissue ontology distance to tissue embedding distance. Tissue ontology distance is defined by adding the shortest path lengths from a pair of tissue nodes to the lowest common ancestor node in the tissue hierarchy, and tissue embedding distance is the cosine distance between the representations of a pair of tissue nodes. We expect a positive correlation: the farther apart the nodes are according to the tissue hierarchy, the more dissimilar the tissue representations are. However, we do not expect a correlation coefficient of 1 since the tissue representations capture additional information not present in the tissue ontology. The Pinnacle tissue embedding distances are non-random (Kolmogorov-Smirnov two-sided test = 0.50; *p*-value < 0.001). As hypothesized, there is a positive correlation between tissue embedding and ontology distances (Spearman’s *ρ* = 0.36; *p*-value = 1.85 × 10^−119^; Figure 3c). In contrast, there is no correlation when the tissue ontology is shuffled (Spearman’s *ρ* = 0.005; *p*-value = 0.349; Figure 3c), indicating that Pinnacle’s tissue representations indeed reflect the tissue organization. Since tissue organization is propagated throughout cell type and protein representations of Pinnacle, all of Pinnacle’s representations capture tissue organization (Methods 3; Supplementary Figure S6).

### Context improves structural prediction of PD-1/PD-L1 and B7-1/CTLA-4 protein binders.

Protein-protein interactions (PPI) are often dependent on both structural conformations of the proteins [70, 71] and their environment, such as cell type context [72]. Protein representations produced on the basis of 3D molecular structures of proteins lack cell type context information in which proteins act. Considering alternative conformations of proteins has yielded improvements in binding predictions [70]. We hypothesize that incorporating cellular context information can better differentiate binding from non-binding proteins (Figure 3d). As there is no systematically generated dataset with matched structural biology and genomic readouts to perform a large-scale analysis, we focus on two pairs of interacting proteins, PD-1/PD-L1 and B7-1/CTLA-4. We aim to establish through these two case studies a connection between context-specific representations learned from PPI networks and 3D structure-based representations derived from structural biology.

We compare contextualized and context-free protein representations for binding proteins (i.e., PD-1/PD-L1 and B7-1/CTLA-4) and non-binding proteins (i.e., one of the four binding proteins paired with RalB, RalBP1, EPO, EPOR, C3, or CFH). Cell type context is incorporated into 3D structure-based protein representations [10, 43] by concatenating them with Pinnacle’s protein representation (Figure 3e; Methods 4). Context-free protein representations are generated by concatenating 3D structure-based representations [10, 43] with an average of Pinnacle’s protein representations across all cell type contexts (Methods 4). Using context-free representations, binding and non-binding proteins are scored (via cosine similarity) at 0.9789 ± 0.0004 and 0.9742 ± 0.0078, respectively. In contrast, with Pinnacle’s context-aware protein representations, which have no knowledge of 3D structure, binding and non-binding proteins are scored 0.0385 ± 0.1531 and 0.0218 ± 0.1081, respectively. Contextualized representations, resulting from a combination of protein representations based on 3D structure and context-aware PPI networks, give scores for binding and non-binding proteins of 0.9690 ± 0.0049 and 0.9571 ± 0.0127, respectively. Comparative analysis of the gap in scores between binders and non-binders yields gaps of 0.011 (PD-1/PD-L1) and 0.015 (B7-1/CTLA-4) for contextualized representations, yet only 0.003 (PD-1/PD-L1) and 0.006 (B7-1/CTLA-4) for context-free representations (Figure 3f). Incorporating information about biological contexts can help better distinguish protein binders (namely, PD-1/PD-L1 and B7-1/CTLA-4) from non-binders in specific cell types, suggesting that Pinnacle’s context-aware representations have the potential to enhance protein representations derived from complementary 3D structure data modality. Modeling context specific binding of PD-1/PD-L1 and B7-1/CTLA-4 may even deepen our understanding of immune checkpoint blockade therapies.

### Nominating therapeutic targets across cell type contexts.

**Figure 4:**
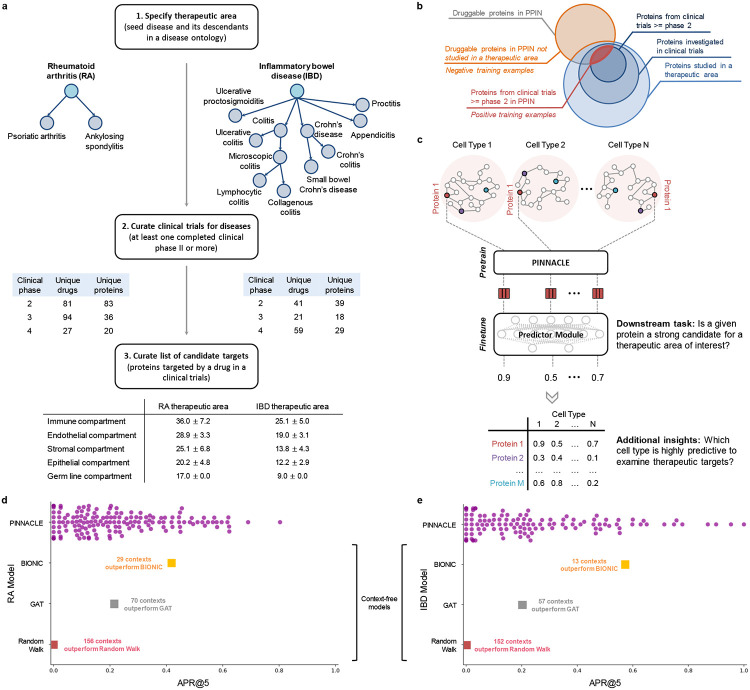
Fine-tuning contextualized protein representations for therapeutic target prioritization. (**a**) Workflow to curate positive training examples for rheumatoid arthritis (left) and inflammatory bowel disease (right) therapeutic areas. (**b**) We construct positive examples by selecting proteins that are targeted by compounds that have at least completed phase 2 for treating the therapeutic area of interest. These proteins are deemed safe and potentially efficacious for humans with the disease. We construct negative examples by selecting proteins that do not have associations with the therapeutic area yet have been targeted by at least one existing drug/compound. (**c**) Cell type-specific protein interaction networks are embedded by Pinnacle, and finetuned for a downstream task. Here, the multi-layer perceptron finetunes the (pretrained) contextualized protein representations for predicting whether a given protein is a strong candidate for the therapeutic area of interest. Additional insights of our setup include hypothesizing highly predictive cell types for examining candidate therapeutic targets. (**d-e**) Benchmarking of context-aware and context-free approaches for (**d**) RA and (**e**) IBD therapeutic areas. Each dot is the performance (averaged across 10 random seeds) of protein representations from a given context (i.e., cell type context for Pinnacle, context-free global reference protein interaction network for GAT and random walk, and context-free multi-modal protein interaction network for BIONIC).

With the representations from Pinnacle that are infused with cellular and tissue context, we can fine-tune them for downstream tasks (Figure 1f-h). We hypothesize that Pinnacle’s contextualized latent space can better differentiate between therapeutic targets and proteins with no therapeutic potential than a context-free latent space. Here, we focus on modeling the therapeutic potential of proteins across cell types for therapeutic areas with cell type-specific mechanisms of action (Figure 4). Certain cell types are known to play crucial and distinct roles in the disease pathogenesis of rheumatoid arthritis (RA) and inflammatory bowel disease (IBD) therapeutic areas [58, 60, 73-81]. There is currently no cure for either types of conditions, and the medications prescribed to mitigate the symptoms can lead to undesired side effects [82-86]. The new generation of therapeutics in development for RA and IBD conditions is designed to target specific cell types so that the drugs maximize efficacy and minimize adverse events (e.g., by directly impacting the affected/responsible cells and avoiding off-target effects on other cells) [84, 87]. We adapt Pinnacle models to predict the therapeutic potential of proteins in a cell type specific manner.

We independently fine-tune Pinnacle to predict therapeutic targets for RA and IBD diseases. Specifically, we perform binary classification on each contextualized protein representation, where y=1 indicates that the protein is a therapeutic candidate for the given therapeutic area and y=0 otherwise. The ground truth positive examples (where y=1) are proteins targeted by drugs that have at least completed one clinical trial of phase two or higher for indications under the therapeutic area of interest, indicating that the drugs are safe and potentially efficacious in an initial cohort of humans (Figure 4a-b). The negative examples (where y=0) are druggable proteins that have not been studied for the therapeutic area (Figure 4b; Methods 5). There is existing evidence that treatment effects vary on the basis of the cell type in which therapeutic targets are expressed [88-92]. For instance, CD19-targeting chimeric antigen receptor T (CAR-T) cell therapy has been highly effective in treating B cell malignancies, yet causes a high incidence of neurotoxicity [90]. A recent study shows that CAR-T cell is inducing off-target effects by targeting the CD19 expressed in brain mural cells, which is likely causing the brain barrier leakiness responsible for neurotoxicity [90]. We hypothesize that the predicted protein druggability varies across cell types, and such variations can provide insights into the cell types’ relevance for a therapeutic area.

**Figure 5:**
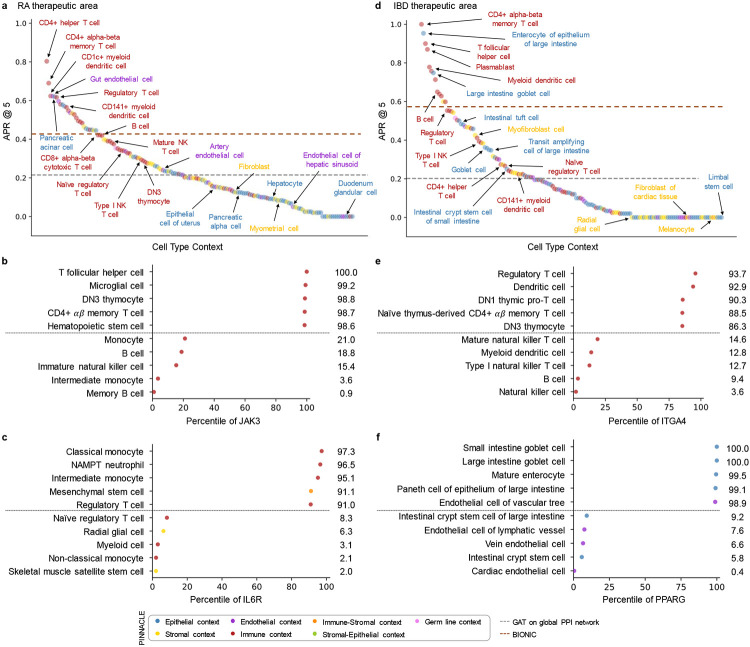
Performance of contextualized target prioritization for RA and IBD therapeutic areas. (**a,d**) Model performance (measured by APR@5) for RA and IBD therapeutic areas, respectively. APR@K (or Average Precision and Recall at K) is a combination of Precision@K and Recall@K (refer to Methods 6 for more details). Each dot is the performance (averaged across 10 random seeds) of Pinnacle’s protein representations from a specific cell type context. The gray and dark orange lines are the performance of the global reference network model and BIONIC model, respectively. For each therapeutic area, 22 cell types are annotated and colored by their compartment category. Supplementary Figure S2 contains model performance measured by APR@10, APR@15, and APR@20 for RA and IBD therapeutic areas. (**b-c, e-f**) Selected proteins for RA and IBD therapeutic areas. Dotted line separates the top and bottom 5 cell types. (**b-c**) Two selected proteins, JAK3 and IL6R, that are targeted by drugs that have completed Phase IV of clinical trials for treating RA therapeutic area. (**e-f**) Two selected proteins, ITGA4 and PPARG, that are targeted by drugs that have completed Phase IV for treating IBD therapeutic area.

Among the 156 biological contexts modeled by Pinnacle’s protein representations, we examine the contexts in which our protein representations are most predictive for RA therapeutic area. We find that the most predictive protein representations are in the context of CD4+ helper T cells, CD4+ *αβ* memory T cells, CD1c+ myeloid dendritic cells, gut endothelial cells, and pancreatic acinar cells (Figure 5a). Immune cells play a significant role in the disease pathogenesis of RA [75, 76]. Since CD4+ helper T cells (Pinnacle-predicted rank = 1), CD4+ *αβ* memory T cells (Pinnacle-predicted rank = 2), and CD1c+ myeloid dendritic cells (Pinnacle-predicted rank = 3) are immune cells, it is expected that Pinnacle’s protein representations in these contexts achieve high performance in our prediction task. Also, patients with RA often have gastrointestinal (GI) manifestations, whether concomitant GI autoimmune diseases or GI side effects of RA treatment [93]. Pancreatic acinar cells (Pinnacle-predicted rank = 5) can behave like inflammatory cells during acute pancreatitis [94], one of the concomitant GI manifestations of RA [93]. In addition to GI manifestations, endothelial dysfunction is commonly detected in patients with RA [95]. While rare, rheumatoid vasculitis, which affects endothelial cells and a serious complication of RA, has been found to manifest in the large and small intestines (gut endothelial cell context has Pinnacle-predicted rank = 4), liver, and gallbladder [93, 96]. Further, many of the implicated cell types for RA patients (e.g., T cells, B cells, natural killer cells, monocytes, myeloid cells, and dendritic cells) are highly ranked by Pinnacle [58-60] (Supplementary Table S1). Our results suggest that injecting cell type context to protein representations can significantly improve performance in nominating therapeutic targets for RA diseases while potentially revealing the cell types underlying disease processes.

The most predictive protein representations for the IBD therapeutic area are in the context of CD4+ *αβ* memory T cells, enterocytes of epithelium of large intestine, T follicular helper cells, plasmablasts, and myeloid dendritic cells (Figure 5d). The intestinal barrier is composed of a thick mucus layer with antimicrobial products, a layer of intestinal epithelial cells, and a layer of mesenchymal cells, dendritic cells, lymphocytes, and macrophages [97]. As such, these five cell types expectedly yield high predictive ability. Moreover, many of the implicated cell types for IBD (e.g., T cells, fibroblasts, goblet cells, enterocytes, monocytes, natural killer cells, B cells, and glial cells) are high ranked by Pinnacle [61, 62, 98] (Supplementary Table S2). For example, CD4+ T cells are known to be the main drivers of IBD [99]. They have been found in the peripheral blood and intestinal mucosa of adult and pediatric IBD patients [100]. Patients with IBD tend to develop uncontrolled inflammatory CD4+ T cell responses, resulting in tissue damage and chronic intestinal inflammation [101, 102]. Due to the heterogeneity of CD4+ T cells in patients, treatment efficacy can depend on the patient’s subtype of CD4+ T cells [101, 102]. Thus, the cell type contexts in which Pinnacle’s protein representations are highly predictive should be further investigated to design safe and efficacious therapies for RA and IBD diseases.

### Pinnacle outperforms context-free models in predicting therapeutic targets.

To evaluate Pinnacle’s contextualized protein representations, we compare Pinnacle’s fine-tuned models against three context-free models. We independently apply a random walk algorithm [103] and a standard graph attention network (GAT) [104] on the context-free reference protein interaction network. The BIONIC model is a graph convolutional neural network designed for (context-free) multi-modal network integration [38]. We find that Pinnacle’s protein representations for all cell type contexts outperform the random walk model for RA (Figure 5a) and IBD (Figure 5d) conditions. Protein representations for 44.9% (70 out of 156) and 37.5% (57 out of 152) of cell types outperform the GAT model for RA (Figure 5a) and IBD (Figure 5d) therapeutic areas, respectively. Although both Pinnacle and BIONIC can integrate the 156 cell type-specific protein interaction networks, Pinnacle’s protein representations for 18.6% of cell types (29 out of 156) and 8.6% of cell types (13 out of 152) outperform BIONIC [38] for RA (Figure 5a) and IBD (Figure 5d), respectively, suggesting that the metagraph’s contribution is not trivial for contextualizing protein representations. Further, Pinnacle outperforms these three context-free models via other metrics for both RA and IBD therapeutic areas (Supplementary Figure S7). We hypothesize that the cell type contexts of protein representations that yield worse performance than the cell type-agnostic protein representations may not have the predictive power (given the current list of targets from drugs that have at least completed phase 2 of clinical trials) for studying the therapeutic effects of candidate targets for RA and IBD therapeutic areas.

In the context-aware model trained to nominate therapeutic targets for RA diseases, the protein representations of duodenum glandular cells, endothelial cells of hepatic sinusoid, myometrial cells, and hepatocytes performance worse than the cell type-agnostic protein representations (Figure 5a). The RA therapeutic area is a group of inflammatory diseases in which immune cells attack the synovial lining cells of joints [75]. Since duodenum glandular cells (Pinnacle-predicted rank = 153), endothelial cells of hepatic sinusoid (Pinnacle-predicted rank = 126), myometrial cells (Pinnacle-predicted rank = 119), and hepatocytes (Pinnacle-predicted rank = 116) are neither immune cells nor found in the synovium, these cell type contexts’ protein representations expectedly perform poorly.

For IBD diseases, the protein representations of the limbal stem cells, melanocytes, fibroblasts of cardiac tissue, and radial glial cells have worse performance than the cell type-agnostic protein representations (Figure 5d). The IBD therapeutic area is a group of inflammatory diseases in which immune cells attack tissues in the digestive tract [78]. As limbal stem cells (Pinnacle-predicted rank = 152), melanocytes (Pinnacle-predicted rank = 147), fibroblasts of cardiac tissue (Pinnacle-predicted rank = 135), and radial glial cells (Pinnacle-predicted rank = 107) are neither immune cells nor found in the digestive tract, these cell type contexts’ protein representations should yield worse performance than context-free representations.

The least predictive cellular contexts in Pinnacle’s models for RA and IBD have no known role in disease, indicating that protein representations from these cell type contexts are poor predictors of RA and IBD therapeutic targets. Pinnacle’s overall improved predictive ability compared to context-free models indicates the importance of understanding cell type contexts in which therapeutic targets are expressed and act.

### Predictive cell type contexts reflect mechanisms of action for RA therapies.

Recognizing and leveraging the most predictive cell type context for examining a therapeutic area can be beneficial for predicting candidate therapeutic targets [88-92]. We find that considering only the most predictive cell type contexts can yield significant performance improvements compared to context-free models (Supplementary Figure S8). To further illustrate the utility of the hypotheses generated by our models, we examine the most predictive cell type contexts of two protein targets for widely used treatments that mitigate the symptoms of RA: JAK3 and IL6R.

Disease-modifying anti-rheumatic drugs (DMARDs), such as Janus kinase (JAK) inhibitors (i.e., tofacitinib, upadacitinib, and baricitinib), are commonly prescribed to patients with RA [105, 106]. For JAK3, Pinnacle’s five most predictive cell type contexts are T follicular helper cells, microglial cells, DN3 thymocytes, CD4+ *αβ* memory T cells, and hematopoietic stem cells (Figure 5b). Since the expression of JAK3 is limited to hematopoietic cells, mutations or deletions in JAK3 tend to cause defects in T cells, B cells, and NK cells [107-110]. For instance, patients with JAK3 mutations tend to be depleted of T cells [108], and the abundance of T follicular helper cells is highly correlated with RA severity and progression [111]. JAK3 is also highly expressed in double negative (DN) T cells (early stage of thymocyte differentiation) [112], and the levels of DN T cells are higher in synovial fluid than peripheral blood, suggesting a possible role of DN T cell subsets in RA pathogenesis [113]. Lastly, dysregulation of the JAK/STAT pathway, which JAK3 participates in, has pathological implications for neuroinflammatory diseases, a significant component of disease pathophysiology in RA [114, 115].

Tocilizumab and sarilumab are FDA approved for the treatment of RA and target the interleukin six receptor, IL6R [106]. For IL6R, Pinnacle’s five most predictive cellular contexts are classical monocytes, NAMPT neutrophils, intermediate monocytes, mesenchymal stem cells, and regulatory T cells (Figure 5c). IL6R is predominantly expressed on neutrophils, monocytes, hepatocytes, macrophages, and some lymphocytes [116]. IL6R simulates the movement of T cells and other immune cells to the site of infection or inflammation [117] and affects T cell and B cell differentiation [116, 118]. IL6 acts directly on neutrophils, essential mediators of inflammation and joint destruction in RA, through membrane-bound IL6R [116]. Experiments on fibroblasts isolated from the synovium of RA patients show that anti-IL6 antibodies prevented neutrophil adhesion, indicating a promising therapeutic direction for IL6R on neutrophils [116]. Lastly, mice studies have shown that pre-treatment of mesenchymal stem/stromal cells with soluble IL6R can enhance the therapeutic effects of mesenchymal stem/stromal cells in arthritis inflammation [119].

Pinnacle’s hypotheses to examine JAK3 and IL6R in the highly predictive cell type contexts, according to Pinnacle, to maximize therapeutic efficacy seems to be consistent with their roles in the cell types. It seems that targeting these proteins may directly impact the pathways contributing to the pathophysiology of RA therapeutic areas. Further, our results for IL6R suggest that Pinnacle’s contextualized representations could be leveraged to evaluate potential enhancement in efficacy (e.g., targeting multiple points in a pathway of interest).

### Predictive power of cell type contexts elucidates the mechanisms of action for IDB therapies.

Similar to RA therapeutic area, we must understand the cells in which therapeutic targets are expressed and act to maximize the efficacy of molecular and cellular therapies for IBD therapeutic area [120]. To support our hypothesis, we evaluate Pinnacle’s predictions for two protein targets of commonly prescribed treatments for IBD diseases: ITGA4 and PPARG.

Vedolizumab and natalizumab target the integrin subunit alpha 4, ITGA4, to treat the symptoms of IBD therapeutic area [106]. Pinnacle’s five most predictive cell type contexts for ITGA4 are regulatory T cells, dendritic cells, DN1 thymic pro-T cells, naive thymus-derived CD4+ *αβ* memory T cells, and DN3 thymocytes (Figure 5e). Integrins mediate the trafficking and retention of immune cells to the gastrointestinal tract; immune activation of integrin genes, such as ITGA4, increases the risk of IBD [121]. For instance, ITGA4 is involved in homing memory and effector T cells to inflamed tissues, including intestinal and non-intestinal tissues, and imbalances in regulatory and effector T cells may lead to inflammation [122]. The homing of CD4+ T cells to the gut lamina propria also depends on integrins encoded by ITGA4 [123]. Circulating dendritic cells express the gut homing marker encoded by ITGA4 as well; the migration of blood dendritic cells to the intestine leads these dendritic cells to become mature, activated, and contribute to gut inflammation and tissue damage, indicating that future studies are warranted to elucidate the functional properties of blood dendritic cells in IBD [124].

Balsalazide and mesalamine are aminosalicylate drugs (DMARDs) commonly used to treat ulcerative colitis by targeting peroxisome proliferator activated receptor gamma (PPARG) [106, 125]. Pinnacle’s five most predictive cell types for PPARG are goblet cells of small and large intestines, mature enterocytes, paneth cells of the epithelium of large intestines, and endothelial cells of the vascular tree (Figure 5f). PPARG is highly expressed in the gastrointestinal tract, higher in the large intestine (e.g., colonic epithelial cells) than the small intestine [126-128]. In patients with ulcerative colitis, PPARG is often substantially downregulated in their colonic epithelial cells [128]. PPARG promotes enterocyte development [129] and intestinal mucus integrity by increasing the abundance of goblet cells [128]. Further, PPARG activation can inhibit endothelial inflammation in vascular endothelial cells [130, 131], which is significant due to the importance of vascular involvement in IBD [132]. The predictive power of cell type contexts to examine ITGA4 and PPARG, according to Pinnacle, for IBD therapeutic development are thus well-supported.

## Discussion

Pinnacle is a flexible geometric deep learning approach for contextualized prediction in user-defined biological contexts. Integrating single-cell transcriptomic atlases with the protein interactome, cell type interactions, and tissue hierarchy, Pinnacle produces latent protein representations specialized to each biological context. Pinnacle’s protein representations capture cellular and tissue organization spanning 156 cell types and 62 tissues of varying hierarchical scales. In addition to multi-modal data integration, a pretrained Pinnacle model generates protein representations that can be used for downstream prediction for tasks where cell type dependencies and cell type-specific mechanisms are important.

One limitation of the study is the use of the human protein interactome, which is not measured in a cell type-specific manner [1]. No systematic measurements of protein interactions across cell types exist. So, we create cell type-specific protein interaction networks by overlaying single-cell measurements on the reference protein interaction network, leveraging previously validated techniques for the reconstruction of cell-type-specific interactomes at single-cell resolution [35] and conducting sensitivity network analyses to confirm the validity of the networks used to train Pinnacle models (Supplementary Figures S1-S2). This approach enriches networks for cell type-relevant proteins (Supplementary Figure S1). The resulting networks may contain false-positive protein interactions (e.g., proteins that interact in the reference protein interaction network but do not interact in a specific cell type) and false-negative protein interactions (e.g., proteins that interact only within a particular cell type context that may be unknown, or even undiscovered). Nevertheless, strong performance gains of Pinnacle over context-free models indicate the importance of contextualized prediction and suggest a direction to enhance existing analyses on protein interaction networks [6, 16, 20, 22, 23, 52, 133, 134].

We can leverage and extend Pinnacle in many ways. Pinnacle can accommodate and supplement diverse data modalities. We developed Pinnacle models using Tabula Sapiens [46], a molecular reference atlas comprising almost 500,000 cells from 24 distinct tissues and organs. However, since the tissues and cell types associated with specific diseases may not be entirely represented in the atlas of healthy human subjects, we anticipate that our predictive power may be limited. Tabula Sapiens does not include synovial tissues associated with RA disease progression [59, 60], but these can be found in synovial RA atlases [135, 136] and stromal cells obtained from individuals with chronic inflammatory diseases [137]. To enhance the predictive ability of Pinnacle models, they can be trained on disease-specific or perturbation-specific networks [138-140]. In addition to using physical protein contacts, Pinnacle can also be applied to cell type-specific protein networks created from other modalities, such as cell type-specific gene expression networks [141-143]. Furthermore, we show that Pinnacle’s representations can supplement protein representations generated from other data modalities, including protein molecular surfaces [10, 43]. While this study is concentrated on protein-coding genes, information on protein isoforms and differential information, such as alternative splicing or allosteric changes, can be used with Pinnacle when such data become available. Also, in this study, Pinnacle representations capture physical interactions between proteins at the cell type level. These representations can be combined with representations produced from protein sequences and structures to generate higher resolution predictions [144]. Lastly, in addition to prioritizing potential therapeutic protein targets, Pinnacle’s representations can be fine-tuned to identify populations of cells with specific characteristics, such as drug resistance [145], adverse drug events [140], or disease progression biomarkers [139].

Prevailing protein representation learning models are context-free, limiting their use to analyze molecular phenotypes that are resolved by contexts and vary with cell types and tissues. To address this limitation, we introduce a contextual learning approach that produces specialized protein representations tailored to biological contexts and demonstrate that such contextual modeling has implications for precision biology [146]. Using contextual learning, we could improve the accuracy of predicting drug effects and treatment response by leveraging cell type dependencies. As experimental and computational technologies advance, it is possible to more accurately model protein representations across different cell type contexts to identify cell type-specific biomarkers and therapeutic candidates [147].

## Online Methods

The Methods describe (1) the curation of datasets, (2) the construction and representation of multi-scale single-cell networks, (3) our multi-scale graph neural network, Pinnacle, (4) the finetuning of Pinnacle for target prioritization, and (5) the metrics and statistical analyses used.

### 1 Datasets

#### Reference human physical protein interaction network.

Our global reference protein-protein interaction (PPI) network is the union of physical multi-validated interactions from BioGRID [148, 149], the Human Reference Interactome (HuRI) [1], and Menche *et al.* [133] with 15,461 nodes and 207,641 edges. Different sources of PPI have their own methods of curating and validating physical interactions between proteins. BioGRID, HuRI, and Menche *et al.* are PPI networks from three well-cited publications and databases regarding human protein interactions. By joining the three networks, we construct a comprehensive global PPI for our analysis.

#### Multi-organ, single-cell transcriptomic atlas of humans.

We leverage Tabula Sapiens [46] data source for our multi-organ, single-cell transcriptomic atlas of humans. The data consists of 15 donors, with 59 specimens total. There are 483,152 cells after quality control, of which 264,824 are immune cells, 104,148 are epithelial cells, 31,691 are endothelial cells, and 82,478 are stromal cells. The cells correspond to 177 unique cell ontology classes.

### 2 Construction of multi-scale networks

Our multi-scale networks comprise protein-level physical interactions, cell-cell communication, cell type-tissue relationships, and tissue-tissue hierarchy.

#### Cell type-specific protein interaction networks.

For each cell type, we create a cell type specific network that represents the physical interactions between proteins (or genes) that are likely expressed in the cell type. Intuitively, our approach identifies genes significantly expressed in a given cell type with respect to the rest of the cells in the dataset. Concretely, we use the processed Tabula Sapiens count matrix to calculate the average expression of each gene in a cell type of interest and the average expression of the corresponding gene in all other cells. Then, we use the Wilcoxon rank-sum test on the two sets of average gene expression. From the resulting ranked list of genes based on activation, we filter for the top *K* most activated genes. We repeat these two steps *N* times and filter for genes that appear in at least 90% of iterations. Finally, we extract these genes’ corresponding proteins from the global protein interaction network and take only the largest connected component. To ensure high-quality representations of cell types in our networks, we keep networks with at least 1,000 proteins. We do not perform subsampling of cells (i.e., sample the same number of cells per cell type) to minimize information loss for constructing protein interaction networks (Supplementary Figure S1). Limitations are described in the Discussion section.

#### Cell type and tissue relationships in the metagraph.

We identify cell-cell interactions based on ligand-receptor (LR) expression using the CellPhoneDB [67] tool and database. An edge between a pair of cell types indicates that CellphoneDB predicts at least one significantly expressed LR pair (with a *p*-value of less than 0.001) between them. To ensure that we have high-quality cell type-cell type interactions, we run CellPhoneDB for 100 iterations. We retain a cell type-cell type interaction if significantly expressed LR pairs (i.e., at least one with a *p*-value of less than 0.001) exist in at least 90 iterations. We determine cell-tissue relationships and extract tissue-tissue relationships using Tabula Sapiens meta-data. For cell-tissue relationships, we draw edges between cells and the tissue they were taken from. For tissue-tissue relationships, we select the nodes corresponding to the tissues where samples were taken from and all parent nodes up to the root of the BRENDA tissue ontology [68].

#### Final dataset.

We have 156 cell type specific protein interaction networks, which have, on average, 2, 530.5 ± 676.8 proteins per network. The number of unique proteins across all cell type specific protein interaction networks is 13, 643 of the 15, 461 proteins in the global reference protein interaction network. In the metagraph, we have 62 tissues (nodes), and 24 are directly connected to cell types. There are 3,567 cell-cell interactions, 372 cell-tissue edges, and 79 tissue-tissue edges.

### 3 Multi-scale graph neural network

#### Overview.

Pinnacle performs biologically-informed message passing through proteins, cell types, and tissues to learn cell type specific protein representations, cell type representations, and tissue representations in a unified multi-scale embedding space. Specifically, Pinnacle traverses through protein-protein physical interactions in each cell type specific PPI network, cell type-cell type communication, cell type-tissue relationships, and tissue-tissue hierarchy with an attention mechanism over individual nodes and edge types. Its objective function is designed and optimized for learning the topology across biological scales, from proteins to cell types to tissues. The resulting embeddings from Pinnacle can be visualized and manipulated for hypothesis-driven interrogation and finetuned for diverse downstream biomedical prediction tasks.

#### Problem Formulation.

Let $𝒢=\{G_{1},\ldots ,G_{|𝒞|}\}$ be a set of cell type specific protein-protein interaction networks, where 𝒞 is a set of unique cell types. Each $G_{c_{i}}=(V_{c_{i}},E_{c_{i}})$ consists of a set of nodes $V_{c_{i}}$ and edges $E_{c_{i}}$ for a given cell type $c_{i}\in 𝒞$ specific protein-protein interaction network. Their nodes v, $v\in V_{c_{i}}$ are proteins, and edges $e_{u,v}^{\text{PP}}\in E_{c_{i}}$ are physical protein-protein interactions (denoted with PP in the superscript). Cell types and tissues form a network, referred to as a metagraph. The metagraph’s set of nodes comprises cell types $c_{i}\in 𝒞$ and tissue $t_{i}\in 𝒯$. The types of edges are cell type-cell type interactions (denoted with CC in the superscript) $e_{c_{i},c_{j}}^{\text{CC}}$ between any pair of cell types $c_{i}$, $c_{j}\in 𝒞$; cell type-tissue associations (denoted with CT in the superscript) $e_{c_{i},t_{i}}^{\text{CT}}$ between any pair of cell type $c_{i}\in 𝒞$ and tissue $t_{i}\in 𝒯$; and tissue-tissue relationships (denoted with TT in the superscript) $e_{t_{i},t_{j}}^{\text{TT}}$ between any pair of tissues $t_{i}$, $t_{j}\in 𝒯$.

#### 3.1 Protein-level attention with cell type specificity

For each cell type specific protein-protein interaction network ${𝒢}_{c_{i}}$, we leverage protein-level attention to learn cell type specific embeddings of proteins. Intuitively, protein-level attention learns which neighboring nodes are likely most important for characterizing a particular cell type’s protein. As such, each cell type specific protein-protein interaction network has its own cell type specific set of learnable parameters. Concretely, at each layer-wise update of layer l of Pinnacle, the node-level attention learns the importance $\alpha_{u,v}$ of protein u to its neighboring protein v in a given cell type $c_{i}\in 𝒞$:

(1) $$h_{u}^{\text{PP}}\leftarrow \text{AGG}(\sigma (\sum_{v\in {𝒩}_{u}}\alpha_{u,v}W^{\text{PP}}h_{v}^{\text{PP}}))$$

where AGG is an aggregation function (i.e., concatenation across K attention heads), σ is the nonlinear activation function (i.e., ReLU), ${𝒩}_{u}$ is the set of neighbors for u (including itself via self-attention), $\alpha_{u,v}$ is an attention mechanism defined as $\alpha_{u,v}=\frac{\text{exp}(\sigma (a^{T}\cdot [h_{u}\|h_{v}]))}{\sum_{v\in {𝒩}_{u}}\text{exp}(\sigma (a^{T}\cdot [h_{u}\|h_{v}]))}$ between a pair of interacting proteins from a specific cell type, $W^{\text{PP}}$ is a PP-specific transformation matrix to project the features of protein u in its cell type specific protein-protein interaction network, and $h_{v}^{\text{PP}}$ is the previous layer’s cell type specific embedding for protein v.

#### 3.2 Metagraph-level attention on cellular interactions and tissue hierarchy

For the metagraph, we use node-level and edge-level attention to learn which neighboring nodes and edge types are likely most important for characterizing the target node (i.e., the node of interest). Intuitively, to learn an embedding for a specific cell type or tissue, we should evaluate the informativeness of each direct cell type or tissue neighbor, as well as the relationship type between the cell type or tissue and their neighbors (e.g., parent-child tissue relationship, tissue from which a cell type is found, cell type with which the cell type of interest communicates with).

Concretely, at each layer l of Pinnacle, the embeddings of a cell type $c_{i}\in 𝒞$ are the result of aggregating the embeddings, $h_{c}^{\text{CC}}$ and $h_{t}^{\text{CT}}$, of its direct cell type neighbor c and tissue neighbor t, that are projected via edge-type-specific transformation matrices, $W^{\text{CC}}$ and $W^{\text{CT}}$, and weighted by learned attention weights, $\alpha_{c_{i},c}$ and $\alpha_{c_{i},t}$ (respectively):

(2) $$h_{c_{i}}^{\text{CC}}\leftarrow \text{AGG}(\sigma (\sum_{c\in {𝒩}_{c_{i}}}\alpha_{c_{i},c}W^{\text{CC}}h_{c}^{\text{CC}}))$$

(3) $$h_{c_{i}}^{\text{CT}}\leftarrow \text{AGG}(\sigma (\sum_{t\in {𝒩}_{c_{i}}}\alpha_{c_{i},t}W^{\text{CT}}h_{c}^{\text{CT}}))$$

The embeddings generated from separately propagating messages through cell type-cell type edges or cell type-tissue edges are combined using learned attention weights $\beta^{\text{CC}}$ and $\beta^{\text{CT}}$, respectively:

(4) $$h_{c_{i}}=\beta^{\text{CC}}h_{c_{i}}^{\text{CC}}+\beta^{\text{CT}}h_{c_{i}}^{\text{CT}}$$

Similarly, the embeddings of a tissue $t_{i}\in 𝒯$ are the result of aggregating the embeddings, $h_{t}^{\text{TT}}$ and $h_{c}^{\text{TC}}$, of its direct tissue neighbor t and cell type neighbor c, that are projected via edge-type-specific transformation matrices, $W^{\text{TT}}$ and $W^{\text{TC}}$, and weighted by learned attention weights, $\alpha_{t_{i},t}$ and $\alpha_{t_{i},c}$ (respectively):

(5) $$h_{t_{i}}^{\text{TT}}\leftarrow \text{AGG}(\sigma (\sum_{t\in {𝒩}_{t_{i}}}\alpha_{t_{i},t}W^{\text{TT}}h_{t}^{\text{TT}}))$$

(6) $$h_{t_{i}}^{\text{TC}}\leftarrow \text{AGG}(\sigma (\sum_{c\in {𝒩}_{t_{i}}}\alpha_{t_{i},c}W^{\text{TC}}h_{c}^{\text{TC}}))$$

The embeddings generated from separately propagating messages through tissue-tissue edges or tissue-cell type edges are combined using learned attention weights $\beta^{\text{TT}}$ and $\beta^{\text{TC}}$, respectively:

(7) $$h_{t_{i}}=\beta^{\text{TT}}h_{t_{i}}^{\text{TT}}+\beta^{\text{TC}}h_{t_{i}}^{\text{TC}}$$

For the node-level attention mechanisms (Equations 2, 3, 5, and 6), AGG is an aggregation function (i.e., concatenation across K attention heads, σ is the nonlinear activation function (i.e., ReLU), ${𝒩}_{c_{i}}$ and ${𝒩}_{t_{i}}$ are the sets of neighbors for $c_{i}$ and $t_{i}$ respectively (includes itself via self-attention), $W^{\text{CC}}$, $W^{\text{CT}}$, $W^{\text{TC}}$, and $W^{\text{TT}}$ are edge-type-specific transformation matrices to project the features of a given target node, $h_{c}^{\text{CC}}$, $h_{t}^{\text{CT}}$, $h_{t}^{\text{TT}}$, and $h_{c}^{\text{TC}}$ are the previous layer’s embedding for c given the edge type CC, t given the edge type CT, t given the edge type TT, and c given the edge type TC, respectively. Finally, the node-level attention mechanism for a given source node u and edge type r is $\alpha_{u,v}^{r}=\frac{\text{exp}(\sigma (a_{r}^{T}\cdot [h_{u}\|h_{v}]))}{\sum_{v\in {𝒩}_{u}}\text{exp}(\sigma (a_{r}^{T}\cdot [h_{u}\|h_{v}]))}$. For the attention mechanisms over edge types (Equations 4 and 7), $\beta^{r}=\frac{\text{exp}(m_{r})}{\sum_{r\in R}\text{exp}(m_{r})}$ such that $m_{r}=\sum_{u\in V_{q}}s^{T}\;\cdot \;\text{tanh}(M\;\cdot \;h_{u}^{r}+b)$ where $V_{q}$ is the set of nodes in the metagraph, s is the attention vector, M is the weight matrix, and b is the bias vector. These parameters are shared for all edge types in the metagraph.

#### 3.3 Bridge between protein and cell type embeddings

Using an up-pooling mechanism, we bridge cell type specific protein embeddings with their corresponding cell type embeddings. We initialize cell type embeddings by taking the average of their proteins’ embeddings: $h_{c_{i}}=\frac{1}{|V_{c_{i}}|}\sum_{u\in V_{c_{i}}}h_{u}$ where $h_{u}$ is the embedding of protein node $u\in V_{c_{i}}$ in the PPI subnetwork for cell type $c_{i}$. Similarly, we initialize tissue embeddings by taking the average of their neighbors: $h_{t_{i}}=\frac{1}{|{𝒩}_{t_{i}}|}\left(\sum_{t\in {𝒩}_{t_{i}}}h_{t}+\sum_{c\in {𝒩}_{t_{i}}}h_{c}\right)$, where $h_{t}$ and $h_{c}$ are the embeddings of tissue node t and cell type node c, respectively, in the immediate neighborhood of source tissue node $t_{i}$. At each layer l>0, we learn the importance $\gamma_{c_{i},u}$ of node $u\in V_{c_{i}}$ to cell type $c_{i}$ such that

(8) $$h_{c_{i}}\leftarrow h_{c_{i}}+\text{AGG}(\sigma (\sum_{u\in V_{c_{i}}}\gamma_{c_{i},u}h_{u}))$$

After propagating cell type and tissue information in the metagraph (namely, Equations 2-6), we apply $\gamma_{c_{i},u}$ to the cell type embedding of $c_{i}$ such that

(9) $$h_{u}\leftarrow h_{u}+\gamma_{c_{i},u}h_{c_{i}}$$

Intuitively, we are imposing the structure of the metagraph onto the PPI subnetworks based on a protein’s importance to its corresponding cell type’s identity.

#### 3.4 Pinnacle: Overall objective function

Pinnacle is optimized for three biological scales: protein-, cell type-, and tissue-level. Concretely, the loss function ℒ has three components corresponding to each biological scale:

(10) $$ℒ={ℒ}_{protein}+(1-\theta )({ℒ}_{celltype}+{ℒ}_{tissue})$$

where ${ℒ}_{protein}$, ${ℒ}_{celltype}$, and ${ℒ}_{tissue}$ minimize the loss from protein-level predictions, cell type-level predictions, and tissue-level predictions, respectively. θ is a tunable parameter with a range of 0 and 1 that scales the contribution of the link prediction loss of the metagraph relative to that of the protein-protein interactions. At the protein level, we consider two aspects: prediction of protein-protein interactions at each cell type specific PPI network $\left({ℒ}_{ppi}\right)$ and prediction of cell type identity of each protein $\left({ℒ}_{celltypeid}\right)$. The contribution of the latter is scaled by λ, which is a tunable parameter with a range of 0 and 1:

(11) $${ℒ}_{protein}=\theta {ℒ}_{ppi}+\lambda {ℒ}_{elltypeid}$$

Intuitively, we aim to simultaneously learn the topology of each cell type specific PPI network (i.e., ${ℒ}_{ppi}$) and the nuanced differences between proteins activated in different cell types. Specifically, we use binary cross-entropy to minimize the error of predicting positive and negative protein-protein interactions in each cell type specific PPI network:

(12) $${ℒ}_{ppi}=\sum_{c_{i}\in 𝒞}\sum_{u,v\in V_{c_{i}}}y_{u,v}\;\log({\hat{y}}_{u,v})+(1-y_{u,v})\;\log(1-{\hat{y}}_{u,v})$$

and center loss [150] for discriminating between protein embeddings $z_{u}$ from different cell types, represented by embeddings denoted as $z_{c_{i}}$:

(13) $${ℒ}_{celltypeid}=\sum_{c_{i}\in 𝒞}\sum_{u\in V_{c_{i}}}\|z_{u}-z_{c_{i}}{\|}_{2}^{2}$$

At the cell type level, we use binary cross-entropy to minimize the error of predicting cell type-cell type interactions and cell type-tissue relationships:

(14) $${ℒ}_{celltype}={ℒ}_{celltype}^{\text{CC}}+{ℒ}_{celltype}^{\text{CT}}$$

such that

(15) $${ℒ}_{celltype}^{\text{CC}}=\sum_{c_{i},c_{j}\in 𝒞}y_{c_{i},c_{j}}\;\log({\hat{y}}_{c_{i},c_{j}})+(1-y_{c_{i},c_{j}})\;\log(1-{\hat{y}}_{c_{i},c_{j}})$$

(16) $${ℒ}_{celltype}^{\text{CT}}=\sum_{c_{i}\in 𝒞}\sum_{t_{k}\in 𝒯}y_{c_{i},t_{k}}\;\log({\hat{y}}_{c_{i},t_{k}})+(1-y_{c_{i},t_{k}})\;\log(1-{\hat{y}}_{c_{i},t_{k}})$$

Similarly, at the tissue level, we use binary cross-entropy to minimize the error of predicting tissue-tissue and tissue-cell type relationships:

(17) $${ℒ}_{tissue}={ℒ}_{tissue}^{\text{TT}}+{ℒ}_{tissue}^{\text{TC}}$$

such that

(18) $${ℒ}_{tissue}^{\text{TT}}=\sum_{t_{k},t_{q}\in 𝒯}y_{t_{k},t_{q}}\;\log({\hat{y}}_{t_{k},t_{q}})+(1-y_{t_{k},t_{q}})\;\log(1-{\hat{y}}_{t_{k},t_{q}})$$

(19) $${ℒ}_{tissue}^{\text{TC}}=\sum_{t_{k}\in 𝒯}\sum_{c_{i}\in 𝒞}y_{t_{k},c_{i}}\;\log({\hat{y}}_{t_{k},c_{i}})+(1-y_{t_{k},c_{i}})\;\log(1-{\hat{y}}_{t_{k},c_{i}})$$

#### 3.5 Training details for Pinnacle

##### Hyperparameter tuning.

We leverage Weights and Biases [151] to select optimal hyperparameters via a random search over the hyperparameter space. The best-performing hyperparameters for Pinnacle are selected by optimizing the ROC and Calinski-Harabasz score [152] on the validation set. The hyperparameter space on which we perform a random search to choose the optimal set of hyperparameters is: the dimension of the nodes’ feature matrix ∈ [1024, 2048], dimension of the output layer ∈ [4, 8, 16, 32], lambda ∈ [0.1, 0.01, 0.001], learning rate for link prediction task ∈ 0.01, 0.001], learning rate for protein’s cell type classification task ∈ [0.1, 0.01, 0.001], number of attention heads ∈ [4, 8], weight decay rate ∈ [0.0001, 0.00001], dropout rate ∈ [0.3, 0.4, 0.5, 0.6, 0.7], and normalization layer ∈ [layernorm, batchnorm, graphnorm, none].

##### Implementation.

We implement Pinnacle using Pytorch (Version 1.12.1) [153] and Pytorch Geometric (Version 2.1.0) [154]. We leverage Weights and Biases [151] for hyperparameter tuning and model training visualization, and we create interactive demos of the model using Gradio [155]. Models are trained on a single NVIDIA Tesla V100-SXM2-16GB GPU.

### 4 Generating contextualized 3D protein representations

After pre-training Pinnacle, we can leverage the output protein representations for diverse downstream tasks. Here, we demonstrate Pinnacle’s ability to improve the prediction of protein-protein interactions by injecting context into 3D molecular structures of proteins.

#### Overview.

Given a protein of interest, we generate both the context-free structure-based representation via MaSIF [10, 43] and a contextualized PPI network-based representation via Pinnacle. We calculate the binding score of a pair of proteins based on either context-free representations or contextualized representations of the proteins. To quantify the added value, if any, provided by contextualizing protein representations with cell type context, we compare the size of the gap between the average binding scores of binding and non-binding proteins in the two approaches.

#### Dataset.

The proteins being compared are PD-1, PD-L1, B7-1, CTLA-4, RalB, RalBP1, EPO, EPOR, C3, and CFH. The pairs of binding proteins are PD-1/PD-L1 (PDB ID: 4ZQK) and B7-1/CTLA-4 (PDB ID: 1I8L). The non-binding proteins are any of the four proteins paired with any of the remaining six proteins (e.g., PD-1/RalB, PD-1/RalBP1, PD-L1/RalBP1). The PDB IDs for the other six proteins are 2KWI for RalB/RalBP1, 1CN4 for EPO/EPOR, and 3OXU for C3/CFH.

#### Structure-based protein representation learning model.

We directly apply the pretrained model for MaSIF [10, 43] to generate the 3D structure-based protein representations. Namely, we use the model pretrained for MaSIF-site, named all_feat_31_seed_benchmark. The output of the model for a given protein is P×d, where P is the number of patches (precomputed by the authors of MaSIF [10, 43]) and d=4 is the dimension of the model’s output layer. As proteins vary in size (i.e., the number of patches to cover the surface of the protein), we select a fixed k number of patches that are most likely to be part of the binding site, according to the pretrained MaSIF model. For each protein, we select k=200 patches, which is the average number of patches for PD-1, PD-L1, B7-1, and CTLA-4, resulting in a matrix of size 200 × 4. Finally, we take the element-wise median on the 200 × 4 matrix to transform it into a vector of length 200. This vector becomes the structure-based protein representation for a given protein.

#### Experimental setup.

For each cell type context of a given protein, we concatenate the 3D structure-based protein representation with the cell type specific protein representation from Pinnacle to generate a contextualized structure-based protein representation. For the context-free protein representation, we concatenate the structure-based protein representation with an element-wise average of Pinnacle’s protein representations. This is to maintain consistent dimensionality between context-free and contextualized protein representations. Given a pair of proteins, we calculate a score via cosine similarity using the context-free or contextualized protein representations. We quantify the gap between the scores of binding and non-binding proteins using context-free or contextualized protein representations.

### 5 Fine-tuning Pinnacle for target prioritization

After pre-training Pinnacle, we can fine-tune the output protein representations for diverse biomedical downstream tasks. Here, we demonstrate Pinnacle’s ability to enhance the performance of predicting a protein’s therapeutic potential for a specific therapeutic area.

#### Overview.

For each protein of interest, we feed its Pinnacle-generated embedding into a multi-layer perceptron (MLP). The model then outputs a score between 0 and 1, where 1 indicates strong candidacy to target (i.e., by a compound/drug) for treating the therapeutic area and 0 otherwise. Since a protein has multiple embeddings corresponding to the cell types it is activated in, the MLP model generates a score for each of the protein’s cell type-specific embeddings (Figure ??a). For example, the embedding of Protein 1 from Cell type 1 is scored independently of the embedding of Protein 1 from Cell type 2. The output scores can be examined to identify the most predictive cell types and the strongest candidates for therapeutic targets in any specific cell type.

#### 5.1 Therapeutic targets dataset

We obtain labels for therapeutic targets from the Open Targets Platform [106].

##### Therapeutic area selection.

To curate target information for a therapeutic area, we examine the drugs indicated for the therapeutic area of interest and its descendants. The two therapeutic areas examined are rheumatoid arthritis (RA) and inflammatory bowel disease. For rheumatoid arthritis, we collected therapeutic data (i.e., targets of drugs indicated for the therapeutic area) from OpenTargets [106] for rheumatoid arthritis (EFO_0000685), ankylosing spondylitis (EFO_0003898), and psoriatic arthritis (EFO_0003778). For inflammatory bowel disease, we collected therapeutic data for ulcerative colitis (EFO_0000729), collagenous colitis (EFO_1001293), colitis (EFO_0003872), proctitis (EFO_0005628), Crohn’s colitis (EFO_0005622), lymphocytic colitis (EFO_1001294), Crohn’s disease (EFO_0000384), microscopic colitis (EFO_1001295), inflammatory bowel disease (EFO_0003767), appendicitis (EFO_0007149), ulcerative proctosigmoiditis (EFO_1001223), and small bowel Crohn’s disease (EFO_0005629).

##### Positive training examples.

We define positive examples (i.e., where the label y=1) as proteins targeted by drugs that have at least completed phase 2 of clinical trials for treating a certain therapeutic area. As such, a protein is a promising candidate if a compound that targets the protein is safe for humans and effective for treating the disease. We retain positive training examples that are activated in at least one cell type specific protein interaction network. The final number of positive training examples for RA and IBD are 152 and 114, respectively.

##### Negative training examples.

We define negative examples (i.e., where the label y=0) as druggable proteins that do not have any known association with the therapeutic area of interest according to Open Targets. A protein is deemed druggable if targeted by at least one existing drug [156]. We extract drugs and their nominal targets from Drugbank [125]. We retain negative training examples that are activated in at least one cell type specific protein interaction network. The final number of negative training examples for RA and IBD are 1,465 and 1,377, respectively.

##### Data processing workflow.

For a therapeutic area of interest, we identify its descendants. With the list of disease terms for the therapeutic area, we curate its positive and negative training examples. We split the dataset such that about 80% of the proteins are in the training set, about 10% of the proteins are in the validation set, and about 10% of the proteins are in the test set. The data splits are consistent for each of the cell type contexts to avoid data leakage.

#### 5.2 Finetuning model details

##### Model architecture.

Our multi-layer perceptron (MLP) comprises an input feedforward neural network, one hidden feedforward neural network layer, and an output feedforward neural network layer. In between each layer, we have a non-linear activation layer. In addition, we use dropout and normalization layers between the input and hidden layer (see the Implementation section for more information). Our objective function is binary cross-entropy loss.

##### Hyperparameter tuning.

We leverage Weights and Biases [151] to select optimal hyperparameters via a random search over the hyperparameter space. The best-performing hyperparameters are selected by optimizing the AUPRC on the validation set. The hyperparameter space on which we perform a random search to choose the optimal set of hyperparameters is the dimension of the first hidden layer ∈ [8, 16, 32], dimension of the second hidden layer ∈ [8, 16, 32], learning rate ∈ [0.01, 0.001, 0.0001], weight decay rate ∈ [0.001, 0.0001, 0.00001, 0.000001], dropout rate ∈ [0.2, 0.3, 0.4, 0.5, 0.6, 0.7, 0.8], normalization layer ∈ [layernorm, batchnorm, none], and the ordering of dropout and normalization layer (i.e., normalization before dropout, or vice versa).

##### Implementation.

We implement the MLP using Pytorch (Version 1.12.1) [153]. In addition, we use Weights and Biases [151] for hyperparameter tuning and model training visualization. Models are trained on a single NVIDIA Tesla M40 GPU.

### 6 Metrics and statistical analyses

Here, we describe metrics, visualization methods, and statistical tests used in our analysis.

#### 6.1 Visualization of embeddings

We visualize Pinnacle’s embeddings using a uniform manifold approximation and projection for dimension reduction (UMAP) [157] and seaborn. Using the python package, umap, we transform Pinnacle’s embeddings to two-dimensional vectors via the parameters: n_neighbors = 10, min_dist = 0.9, n_components = 2, and the euclidean distance metric. The plots are created using the seaborn package’s scatterplot function.

#### 6.2 Visualization of cell type embedding similarity

The pairwise similarity of Pinnacle’s cell type embeddings is calculated using cosine similarity (a function provided by sklearn [158]). Then, these similarity scores are visualized using the seaborn package’s clustermap function. For visualization purposes, similarity scores are mapped to colors after being raised to the 10th power.

#### 6.3 Spatial enrichment analysis of Pinnacle’s protein embeddings

To quantify the spatial enrichment for Pinnacle’s protein embedding regions, we apply a systematic approach, SAFE [69], that identifies regions that are over-represented for a feature of interest (Supplementary Figures S3-S4). The required input data for SAFE are networks and annotations of each node. We first construct an unweighted similarity network on Pinnacle protein embeddings: (1) calculate pairwise cosine similarity, (2) apply a similarity threshold on the similarity matrix to generate an adjacency matrix, and (3) extract the largest connected component. The protein nodes are labeled as 1 if they belong to a given cell type context and 0 otherwise. We then apply SAFE to each network using the recommended settings: neighborhoods are defined using the shortpath_weighted_layout metric for node distance and neighborhood radius of 0.15, and *p*-values are computed using the hypergeometric test without multiple testing correction.

Due to computation and memory constraints, we sample 50 protein embeddings from a cell type context of interest and 10 protein embeddings from each of the other 155 cell type contexts. We use a threshold of 0.3 in our evaluation of Pinnacle’s protein embedding regions (Figure 2; Supplementary Figures S3). We also evaluate the spatial enrichment analysis on networks constructed from different thresholds to ensure that the enrichment is not sensitive to our network construction method: [0.1, 0.2, 0.3, 0.4, 0.5, 0.6, 0.7, 0.8, 0.9] (Supplementary Figures S4). We use the Python implementation of SAFE: https://github.com/baryshnikova-lab/safepy.

#### 6.4 Statistical significance of tissue embedding distance

Tissue embedding distance between a given pair of tissue nodes is calculated using cosine distance (a function provided by sklearn [158]). Tissue ontology distance between a given pair of tissue nodes is calculated by taking the sum of the nodes’ shortest path lengths to the lowest common ancestor (functions provided by networkx [159]. We use the two-sample Kolmogorov-Smirnov test (a function provided by scipy [160]) to compare Pinnacle embedding distances against randomly generated vectors (via the randn function in numpy to sample an equal number of vectors from a standard normal distribution). We also use the Spearman correlation (a function provided by scipy [160]) to correlate Pinnacle embedding distance to tissue ontology distance. We additionally generate a null distribution of tissue ontology distance by calculating tissue ontology distance on a shuffled tissue hierarchy (repeated 10 times). Concretely, we shuffle the node identities of the Brenda Tissue Ontology [68] and compute the pairwise tissue ontology distances.

#### 6.5 Performance metric for therapeutic target prioritization

For our downstream therapeutic target prioritization task, we use a metric called (Average Precision and Recall)@K (APR@K). APR@K leverages a combination of Precision@K and Recall@K to measure the ability to rank the most relevant items (in our case, proteins) among the top K. In essence, APR@K calculates Precision@K for each k∈[1,…,K], multiplying each Precision@k by whether the kth item is relevant, and divides by the total number of relevant items r at K:

$$\text{APR}\;@\;K=\frac{1}{r}\sum_{k=1}^{K}\text{Precision}\;@\;k\cdot \text{rel}(k)$$

where

rel(k)={1,if item atkis relevant0,otherwise}

Given the nature of our target prioritization task, some key advantages of using APR@K include robustness to (1) varied numbers of protein targets activated across cell type-specific protein interaction networks and (2) varied sizes of cell type-specific protein interaction networks.

## Supplementary Material

Supplement 1

## Data Availability

All data used in the paper, including the cell type specific protein interaction networks, the metagraph of cell type and tissue relationships, Pinnacle’s contextualized representations, the therapeutic targets of rheumatoid arthritis and inflammatory bowel diseases, and the final and intermediate results of the analyses, are shared via the project website at https://zitniklab.hms.harvard.edu/projects/PINNACLE.

## Footnotes

1 We provide a HuggingFace Space to explore Pinnacle’s contextualized protein representations.

## Code availability.

Python implementation of the methodology developed and used in the study is available via the project website at https://zitniklab.hms.harvard.edu/projects/PINNACLE. The code to reproduce results, together with documentation and examples of usage, are available on GitHub at https://github.com/mims-harvard/PINNACLE. The interactive demo to explore Pinnacle’s contextualized representations is at https://huggingface.co/spaces/michellemli/PINNACLE.
