# Supplementary material for: Contextualizing protein representations using deep learning on protein networks and single-cell data
Source: bioRxiv. 2023 Jul 19:2023.07.18.549602. Preprint. [Version 1] doi: 10.1101/2023.07.18.549602 (PMC10370131; doi:10.1101/2023.07.18.549602)
Supplement: Supplementary file 1 [file media-1.pdf]

Supplementary Information for

Contextualizing protein representations using deep learning on protein networks and single-cell data

Michelle M. Li[1], Yepeng Huang[1], Marissa Sumathipala[1], Man Qing Liang[1], Alberto Valdeolivas[2], Ashwin N. Ananthakrishnan[1,3], Katherine Liao[1,4], Daniel Marbach[2], and Marinka Zitnik[1,5,6,7‡]

[1]Harvard Medical School, Boston, MA, USA
[2]Roche Pharma Research and Early Development, Pharmaceutical Sciences, Roche Innovation Center Basel, Basel, Switzerland
[3]Division of Gastroenterology, Massachusetts General Hospital, Boston, MA, USA
[4]Division of Rheumatology, Inflammation, and Immunity, Brigham and Women's Hospital, Boston, MA, USA
[5]Kempner Institute for the Study of Natural and Artificial Intelligence, Cambridge, MA, USA
[6]Broad Institute of MIT and Harvard, Cambridge, MA, USA
[7]Harvard Data Science Initiative, Cambridge, MA, USA

‡Corresponding author. Email: marinka@hms.harvard.edu

This PDF file includes:

Supplementary Figures S1 to S8
Supplementary Tables S1 to S2

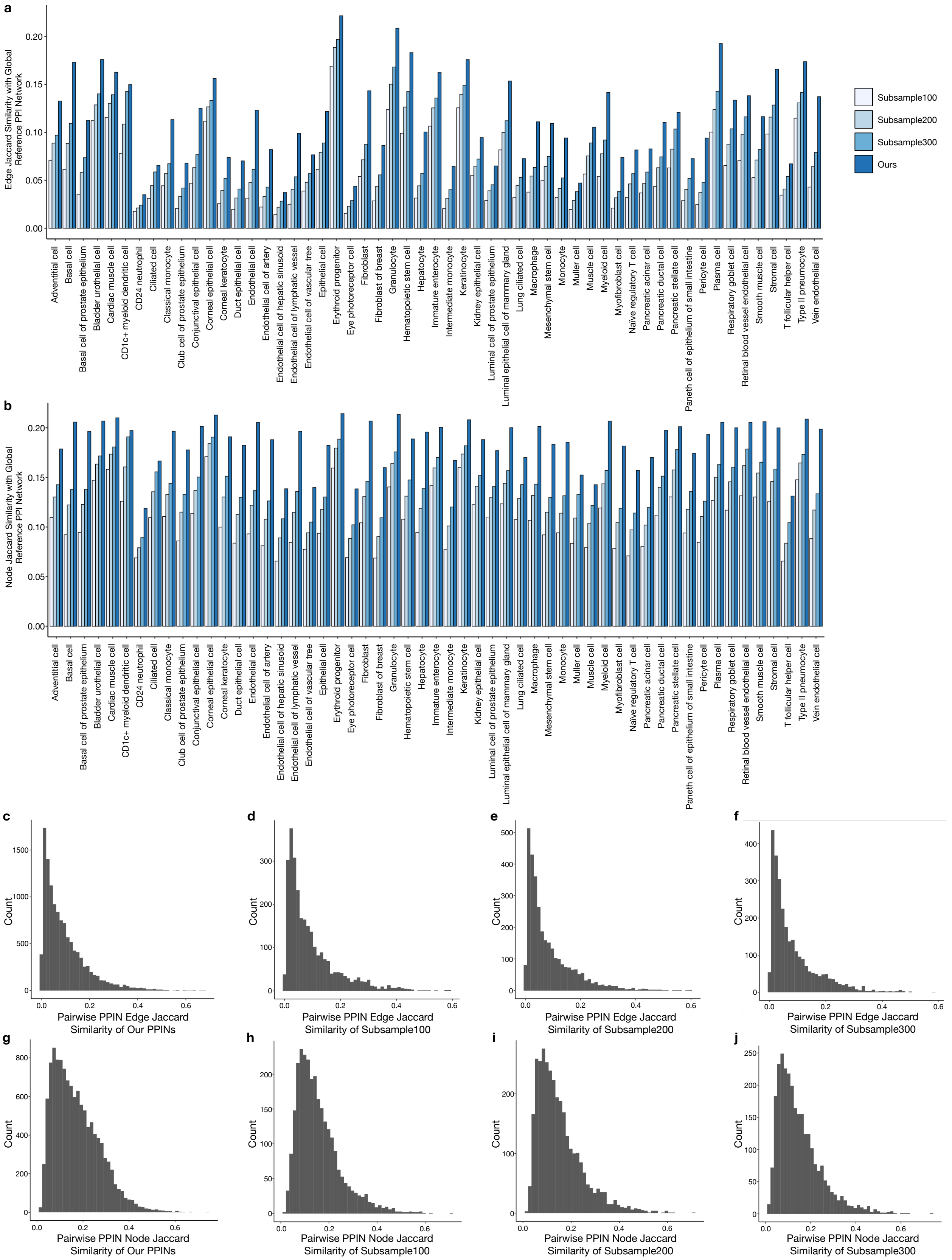

Figure S1: Sensitivity analysis of network construction. To examine whether cell types with fewer cells are poorly represented in our networks, we construct networks after subsampling equal numbers of cells per cell type. We compare our finalized networks (no subsampling of cells) against approaches that subsample 100, 200, and 300 cells. We find that our approach yields networks that are maximally similar to the global reference network yet maintain specificity to cell type context. **(a)** Edge and **(b)** node Jaccard similarity of a cell type-specific PPIN to the global reference PPIN. **(c-j)** Distribution of edge jaccard similarity between PPINs constructed by **(c)** our finalized approach and subsampling **(d)** 100, **(e)** 200, and **(f)** 300 cells. **(g-j)** Distribution of node jaccard similarity between PPINs constructed by **(g)** our finalized approach and subsampling **(h)** 100, **(i)** 200, and **(j)** 300 cells.

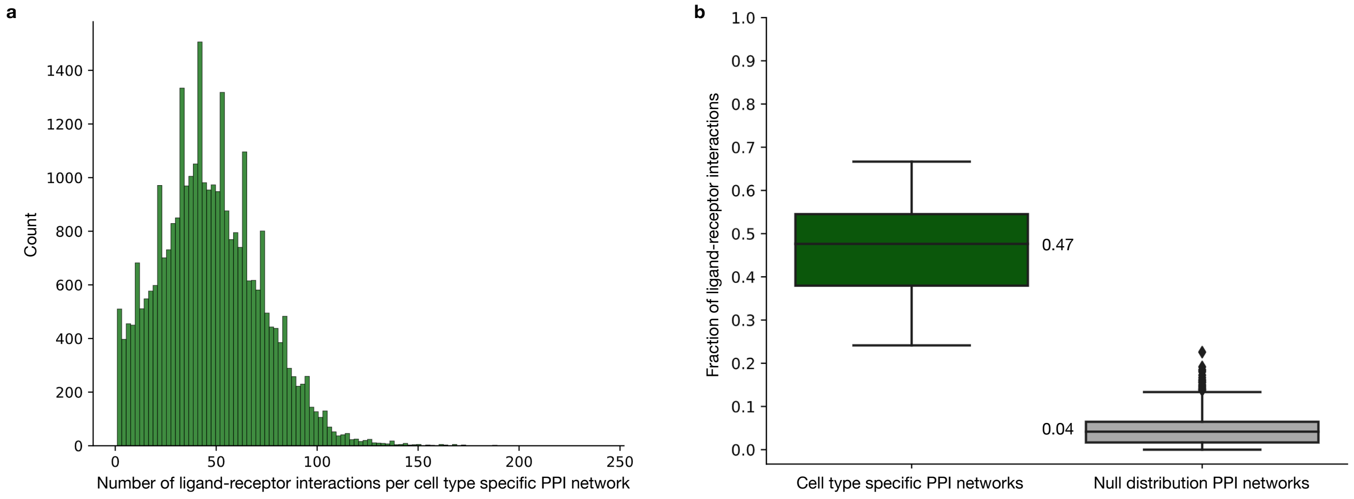

Figure S2: Enrichment analysis of ligand-receptor interactions in the cell type-specific PPI networks. We utilize CellPhoneDB[1] to predict interactions between cell types in our metagraph by identifying significantly expressed ligand-receptor (LR) interactions between pairs of cell types in our dataset. **(a)** Shown is a histogram of the number of significant LR interactions per cell type specific PPI network predicted by CellPhoneDB across 100 iterations. **(b)** We hypothesize that the predicted LR interactions are enriched in our cell type-specific PPI networks. To quantify the enrichment of LR interactions, we calculate the fraction of LR interactions where the corresponding ligand and receptor proteins are activated in the cell type pair (i.e., for a LR interaction identified between cell types A and B, the ligand protein is activated in cell type A's PPI network and the receptor protein is activated in cell type B's PPI network). We compare the fraction of LR pairs that are activated in our cell type-specific PPI networks against the fraction of LR pairs that are activated in null distribution PPI networks. For each cell type-specific PPI network, we generate 100 null distribution PPI networks by sampling the same number of nodes with a similar degree distribution[2]. Degree distribution is preserved by binning nodes such that there are at least 100 nodes in each bin, and nodes are then randomly sampled within the appropriate degree interval[2]. We find that our cell type-specific PPI networks have a significantly higher fraction of ligand-receptor pairs activated (0.47 ± 0.12) than the null distribution PPI networks (0.04 ± 0.04). Note that the ligand-receptor interactions considered in both analyses are those where the genes corresponding to the ligands and receptors are known. However, this does not factor into our construction of the edges/interactions between cell types (CCI).

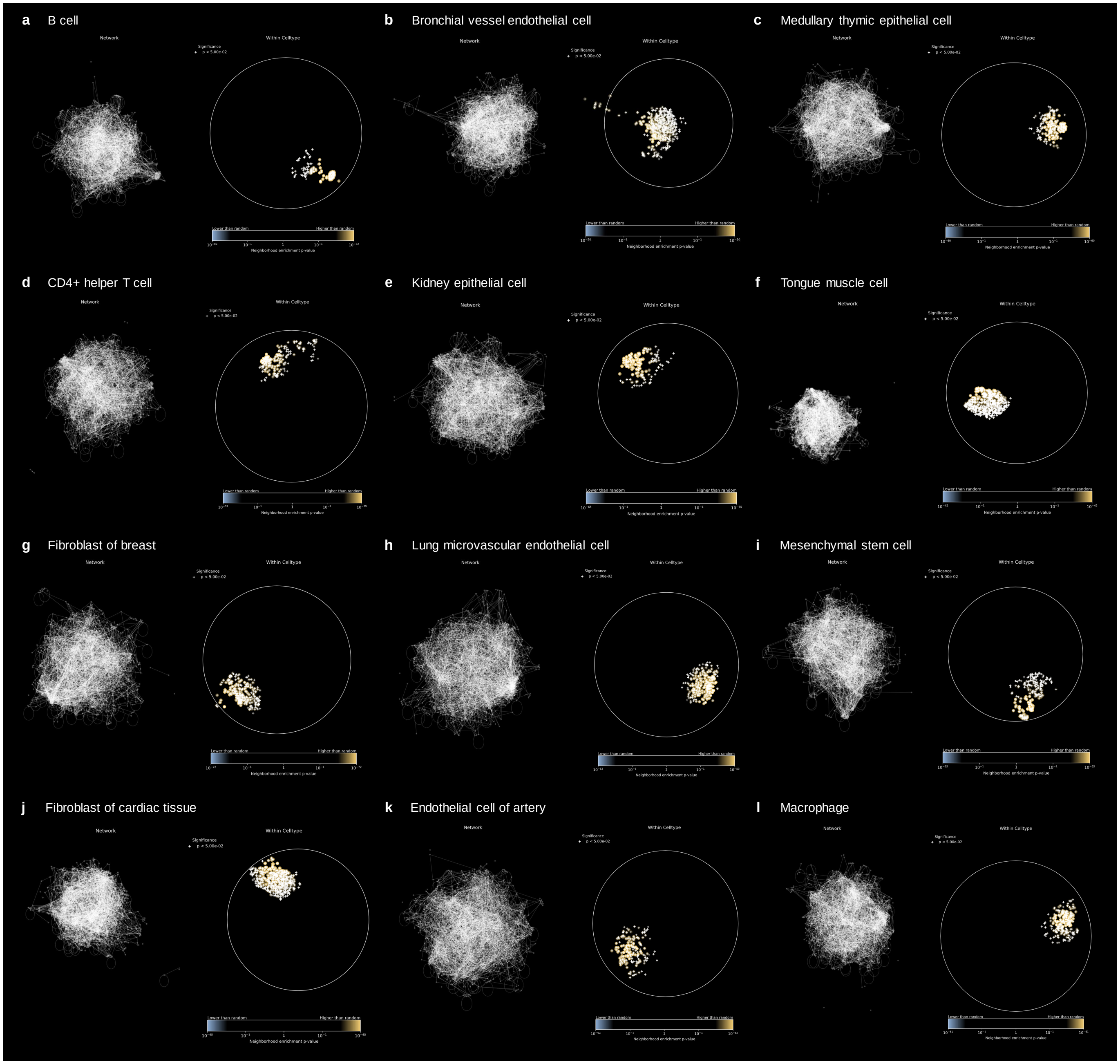

Figure S3: Spatial enrichment analysis of PINNACLE protein embedding regions For each cell type-specific protein embeddings generated by PINNACLE, we sample a subset of PINNACLE protein embeddings to construct a similarity network and perform spatial enrichment analysis using SAFE[3]. Shown for each cell type context is the network (left) and enrichment landscape (right). Dots represent the neighborhood enrichment p-value; crosses indicate a significant p-value < 0.05.

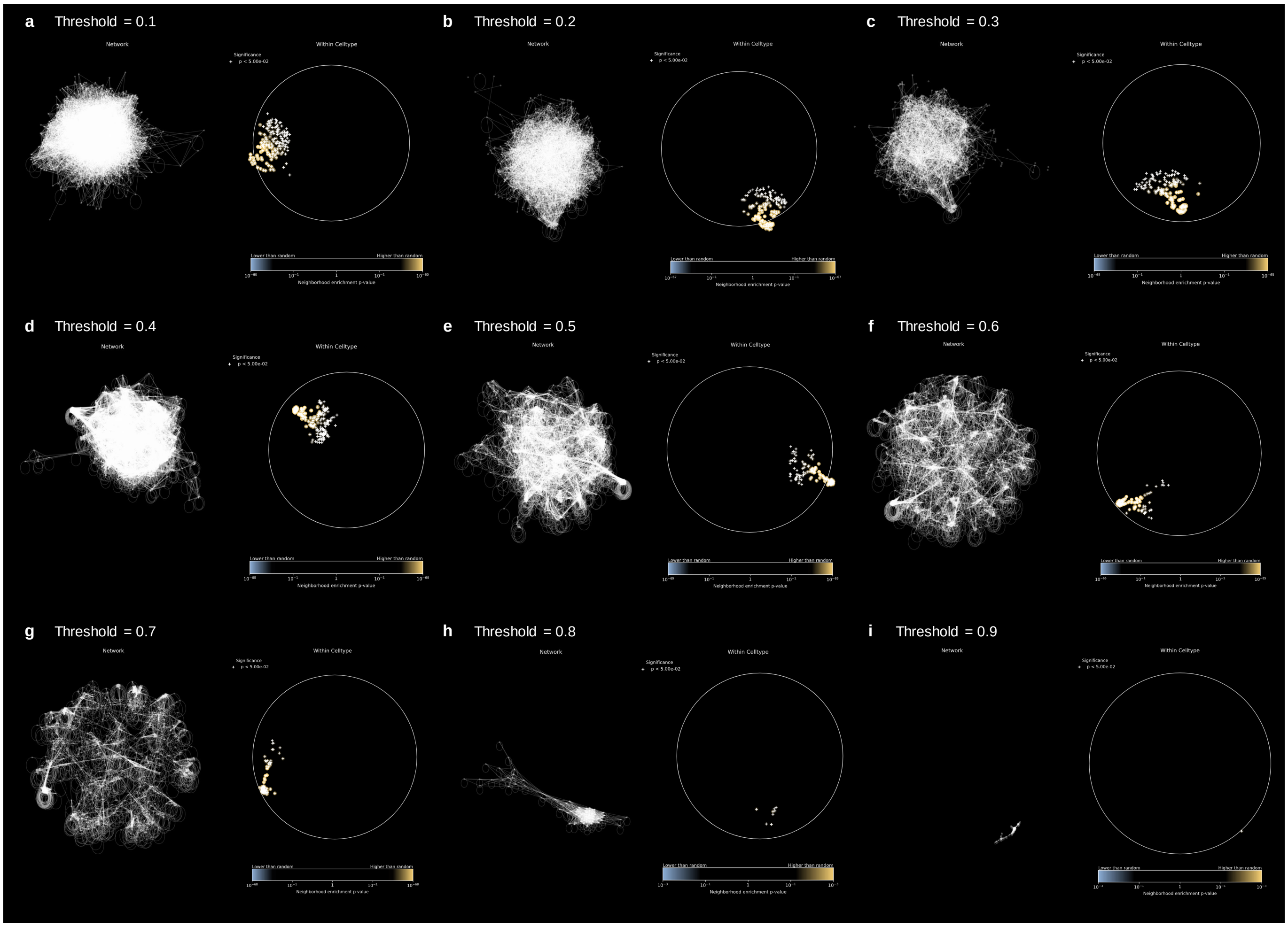

Figure S4: Spatial enrichment analysis of PINNACLE protein embedding regions across thresholds For each cell type-specific protein embeddings generated by PINNACLE, we sample a subset of PINNACLE protein embeddings to construct a similarity network and perform spatial enrichment analysis using SAFE[3]. Networks are constructed using a similarity threshold $t \in [0.1, 0.2, 0.3, 0.4, 0.5, 0.6, 0.7, 0.8, 0.9]$. Shown for each threshold is the network (left) and enrichment landscape (right). Dots represent the neighborhood enrichment p-value; crosses indicate a significant p-value < 0.05.

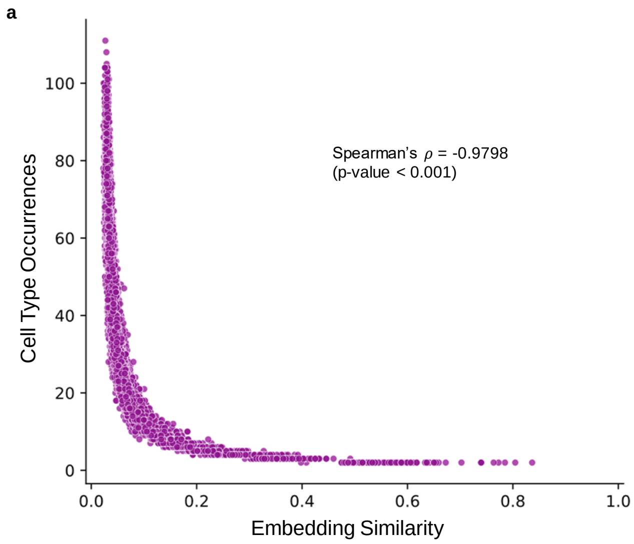

Figure S5: Protein embedding similarity based on cell type activation. Each dot represents a protein that is activated in at least two cell types. Shown is the average cosine similarity of embeddings for each protein as a function of the number of cell types that it is activated in **(a)** with and **(b)** without cellular and tissue context.

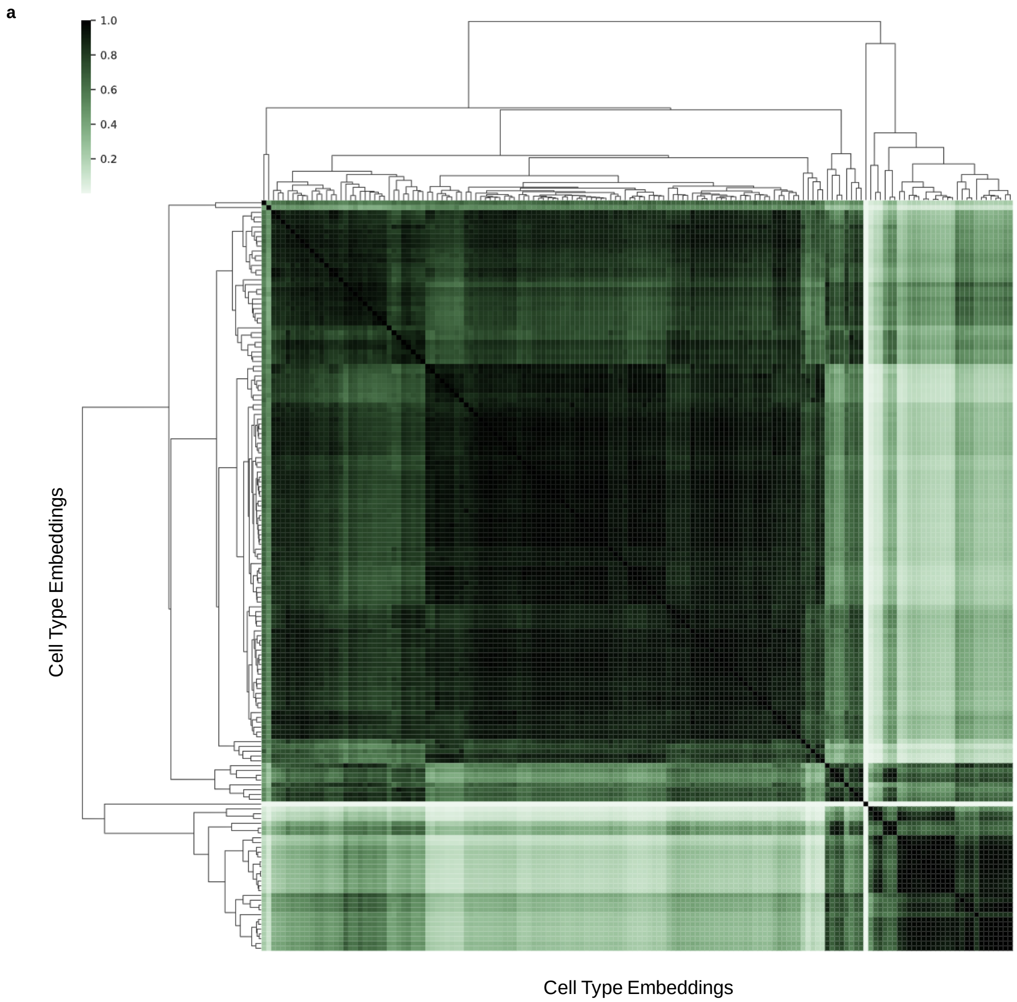

Figure S6: Clustermap of PINNACLE's cell type representations. We quantify the quality of PINNACLE's cell type representations by calculating pairwise similarities of cell type representations. Pairwise similarities are computed via cosine similarity. We expect several major groups of cell type representations that are organized according to cellular and tissue hierarchy and acting as anchors for our complete set of cell type representations. This implies that the contextual information being transferred between the representations of cell types and proteins reflects the tissue hierarchy. Our results show that the local organization of PINNACLE's cell type representations (i.e., identity of cell types in each group) reflects cellular communication, and the global organization of cell type representations (i.e., promixity of groups to each other) reflects tissue organization. Since PINNACLE's protein representations are embedded near their corresponding cell type representation, such organization is enforced among the contextualized protein representations as well.

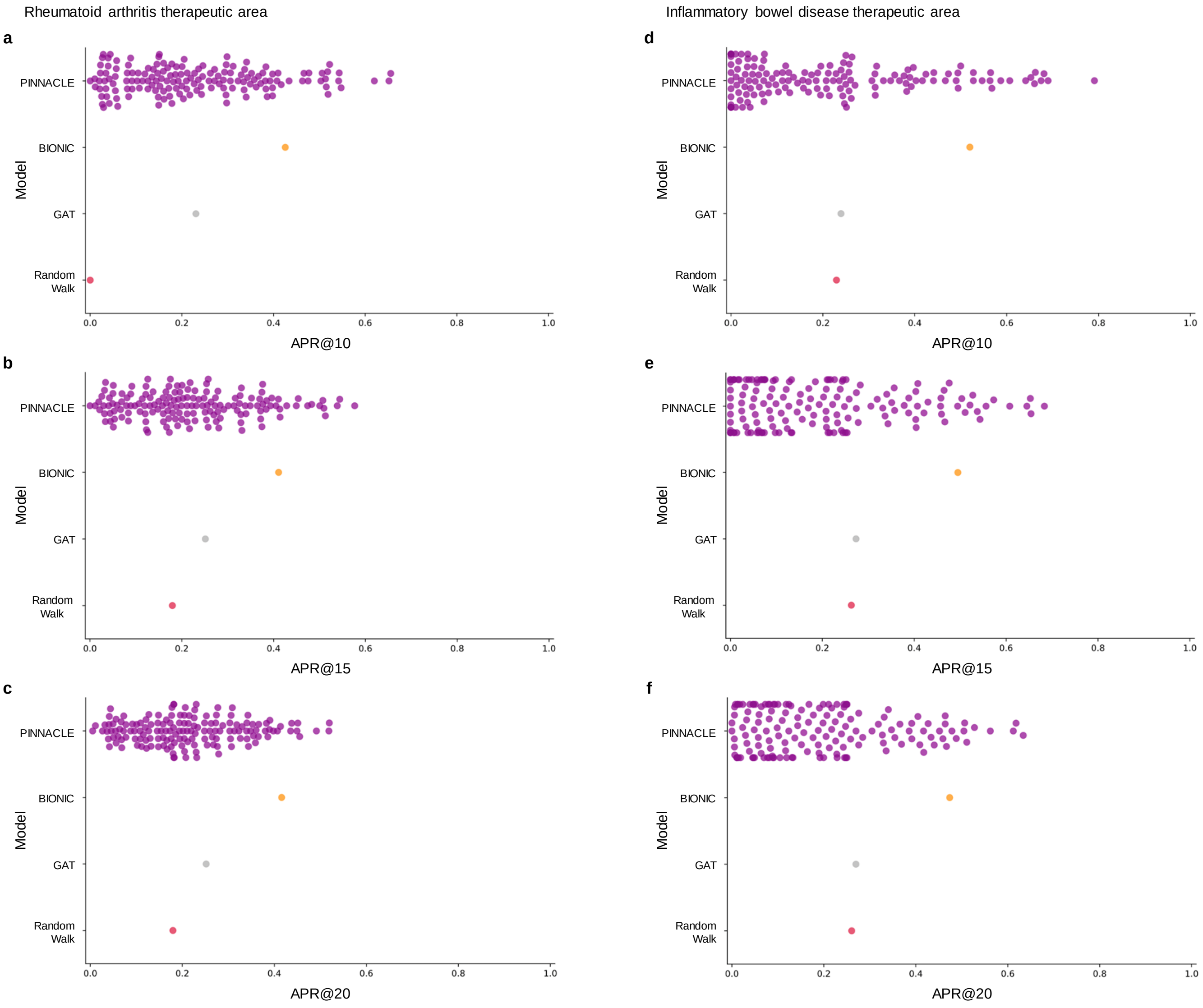

Figure S7: Performance of therapeutic target prioritization models for rheumatoid arthritis and inflammatory bowel disease related conditions. Benchmarking of context-aware and context-free approaches for **(a-c)** RA and **(d-f)** IBD therapeutic areas. Each dot is the performance (averaged across 10 random seeds) of protein representations from a given context (i.e., cell type context for PINNACLE, context-free global reference protein interaction network for GAT and random walk, and context-free multi-modal protein interaction network for BIONIC). In the model for the RA therapeutic area: **(a)** at APR@10, 100% of cell types (156 out of 156) outperform the random walk model, 44.2% of cell types (69 out of 156) outperform the global reference network and 11.5% of cell types (18 out of 156) outperform BIONIC. **(b)** At APR@15, 58.3% (91 out of 156) outperform the random walk model, 38.5% of cell types (60 out of 156) outperform the global reference network and 9.0% of cell types (14 out of 156) outperform BIONIC. **(c)** At APR@20, 59.0 (92 out of 156) outperform the random walk model, 34.6% of cell types (54 out of 156) outperform the global reference network and 5.1% of cell types (8 out of 156) outperform BIONIC. In the model for the IBD therapeutic area: **(d)** at APR@10, 39.5% (60 out of 152) outperform the random walk model, 38.2% of cell types (58 out of 152) outperform the global reference network and 10.5% of cell type (16 out of 152) outperform BIONIC. **(e)** At APR@15, 28.3% (43 out of 152) outperform the random walk model and the global reference network, and 8.6% of cell types (13 out of 152) outperform BIONIC. **(f)** At APR@20, 26.3% (40 out of 152) outperform the random walk model and the global reference network, and 6.6% of cell types (10 out of 152) outperform BIONIC.

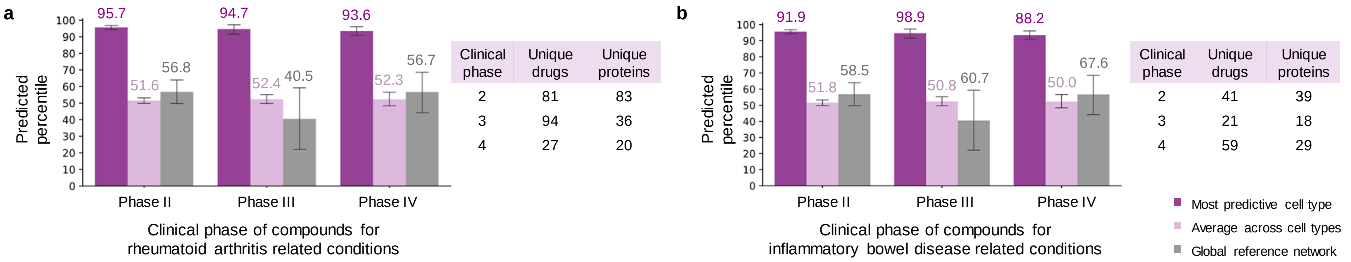

Clinical phase	Unique drugs	Unique proteins
2	81	83
3	94	36
4	27	20

Clinical phase	Unique drugs	Unique proteins
2	41	39
3	21	18
4	59	29

Figure S8: Performance of therapeutic target prioritization models for rheumatoid arthritis and inflammatory bowel disease related conditions stratified by clinical trials. Comparison of the percentiles of drug targets across cell types, in their best-performing cell types, and in the context-free global reference model, stratified by clinical phase of compounds for **(a)** rheumatoid arthritis and **(b)** inflammatory bowel disease related conditions. The table shows the number of unique drugs in each clinical phase, as well as the numbers of unique proteins targeted by those drugs.

Table S1: Predicted ranks of relevant cell types for rheumatoid arthritis related conditions. Shown are predicted ranks of subtypes of T cells, natural killer (NK) cells, dendritic cells, B cells, monocytes, and myeloid cells. These cell types have been demonstrated in existing literature to be involved with rheumatoid arthritis related conditions[4–6].

**Cell type**	**Subtypes**	**PINNACLE-Predicted Rank**
T cell	CD4+ helper T cell	1
	CD4+ $\alpha\beta$ memory T cell	2
	Regulatory T cell	6
	CD8+ $\alpha\beta$ cytotoxic T cell	27
	DN1 thymic pro-T cell	33
	Mature natural killer T cell	34
	Naive regulatory T cell	39
	Type I natural killer T cell	49
	Naive thymus-derived CD4+ $\alpha\beta$ T cell	77
	CD8+ $\alpha\beta$ cytokine secreting effector T cell	104
Dendritic cell	CD1c+ myeloid dendritic cell	3
	CD141+ myeloid dendritic cell	11
	Dendritic cell	37
	Mature conventional dendritic cell	38
	Myeloid dendritic cell	42
	Plasmacytoid dendritic cell	43
	Liver dendritic cell	50
B cell	Memory B cell	12
	B cell	29
Natural killer cell	Natural killer cell	17
	Immature natural killer cell	113
Monocyte	Intermediate monocytes	18
	Non-classical monocytes	68
	Monocyte	85
	Classical monocytes	91
Myeloid cell	Myeloid progenitor	71
	Myeloid cell	95

Table S2: Predicted ranks of relevant cell types for inflammatory bowel disease related conditions. Shown are predicted ranks of subtypes of T cell, fibroblast, goblet cell, enterocyte, monocyte, natural killer cell, B cell, glial cell, dendritic cell, and macrophage. These cell types have been demonstrated in existing literature to be involved with inflammatory bowel disease related conditions[7,8].

Cell type	Subtypes	PINNACLE-Predicted Rank
T cell	CD4+ $\alpha\beta$ memory T cell	1
	Naive thymus-derived CD4+ $\alpha\beta$ T cell	11
	Regulatory T cell	14
	DN1 thymic pro-T cell	15
	Mature natural killer T cell	16
	CD8+ $\alpha\beta$ cytokine secreting effector T cell	19
	Type I natural killer T cell	30
	CD4+ helper T cell	41
	Naive regulatory T cell	43
	CD8+ $\alpha\beta$ cytotoxic T cell	56
Enterocyte	Enterocyte of epithelium of large intestine	2
	Mature enterocyte	22
	Immature enterocyte	90
	Enterocyte of epithelium of small intestine	94
	Intestinal enteroendocrine cell	138
Dendritic cell	Myeloid dendritic cell	5
	Dendritic cell	10
	CD1c+ myeloid dendritic cell	13
	CD141+ myeloid dendritic cell	50
	Liver dendritic cell	54
	Mature conventional dendritic cell	67
	Plasmacytoid dendritic cell	72
Goblet cell	Large intestine goblet cell	7
	Goblet cell	33
	Small intestine goblet cell	75
	Respiratory goblet cell	109
	Tracheal goblet cell	110
B cell	B cell	9
	Memory B cell	45
Monocyte	Intermediate monocyte	26
	Non-classical monocyte	81
	Monocyte	89
	Classical monocyte	93
Glial cell	Microglial cell	29
	Radial glial cell	107
Natural killer cell	Immature natural killer cell	31
	Natural killer cell	73
Fibroblast	Fibroblast	49
Macrophage	Macrophage	74

Supplementary references

1. Efremova, M., Vento-Tormo, M., Teichmann, S. A. & Vento-Tormo, R. Cellphonedb: inferring cell–cell communication from combined expression of multi-subunit ligand–receptor complexes. *Nature Protocols* (2020).

2. Guney, E., Menche, J., Vidal, M. & Barábasi, A.-L. Network-based in silico drug efficacy screening. *Nature Communications* (2016).

3. Baryshnikova, A. Systematic functional annotation and visualization of biological networks. *Cell Systems* (2016).

4. Lewis, M. J. *et al.* Molecular portraits of early rheumatoid arthritis identify clinical and treatment response phenotypes. *Cell Reports* (2019).

5. Zhang, F. *et al.* Cellular deconstruction of inflamed synovium defines diverse inflammatory phenotypes in rheumatoid arthritis. *BioRxiv* (2022).

6. Vickovic, S. *et al.* Three-dimensional spatial transcriptomics uncovers cell type localizations in the human rheumatoid arthritis synovium. *Communications Biology* (2022).

7. Smillie, C. S. *et al.* Intra-and inter-cellular rewiring of the human colon during ulcerative colitis. *Cell* (2019).

8. Kong, L. *et al.* The landscape of immune dysregulation in crohn's disease revealed through single-cell transcriptomic profiling in the ileum and colon. *Immunity* (2023).
